# Supplementary material for: Look-up and look-down neurons in the mouse visual thalamus during freely moving exploration
Source: Curr Biol. 2022 Sep 26;32(18):3987–3999.e4. doi: 10.1016/j.cub.2022.07.049 (PMC9616738; doi:10.1016/j.cub.2022.07.049)
Supplement: Document S1. Figures S1–S5 [file mmc1.pdf]

Current Biology, Volume *32*

Supplemental Information

Look-up and look-down neurons in the mouse visual thalamus during freely moving exploration

Patrycja Orlowska-Feuer, Aghileh S. Ebrahimi, Antonio G. Zippo, Rasmus S. Petersen, Robert J. Lucas, and Riccardo Storchi

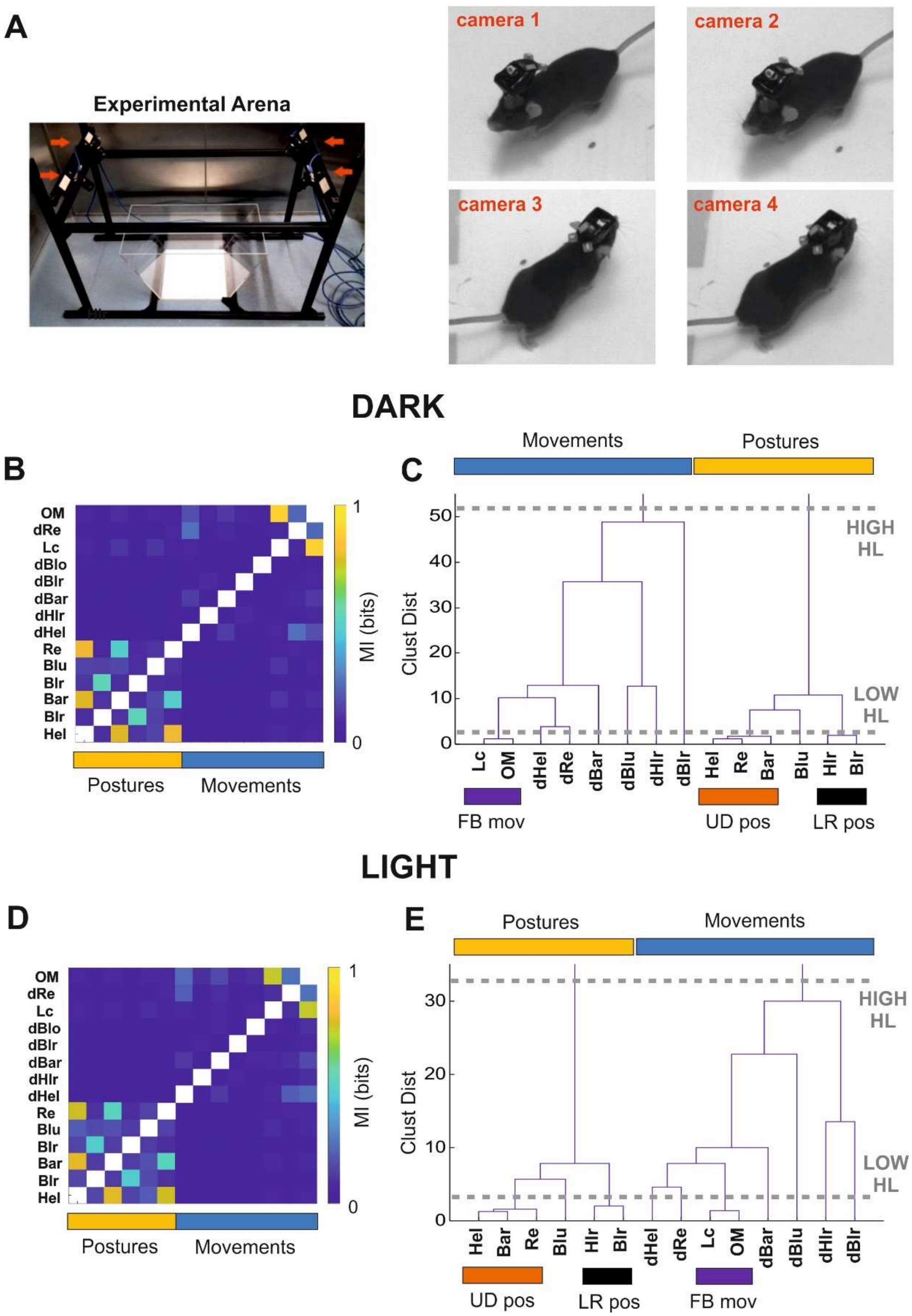

Figure S1. Behavioural set-up and supplemental analyses of behavioural state variables. Related to Figure 1: A) A pictures of the experimental apparatus (left panel). The arena is placed in the centre and imaged by four overhead cameras (indicated by red arrows). A representative frame simultaneously acquired by the 4 cameras is shown (right panel). **B-C)** Same as Figure **1C-D** but for animals recorded in the dark. **D-E)** Same as Figure 1C-D but for animals recorded under photopic light.

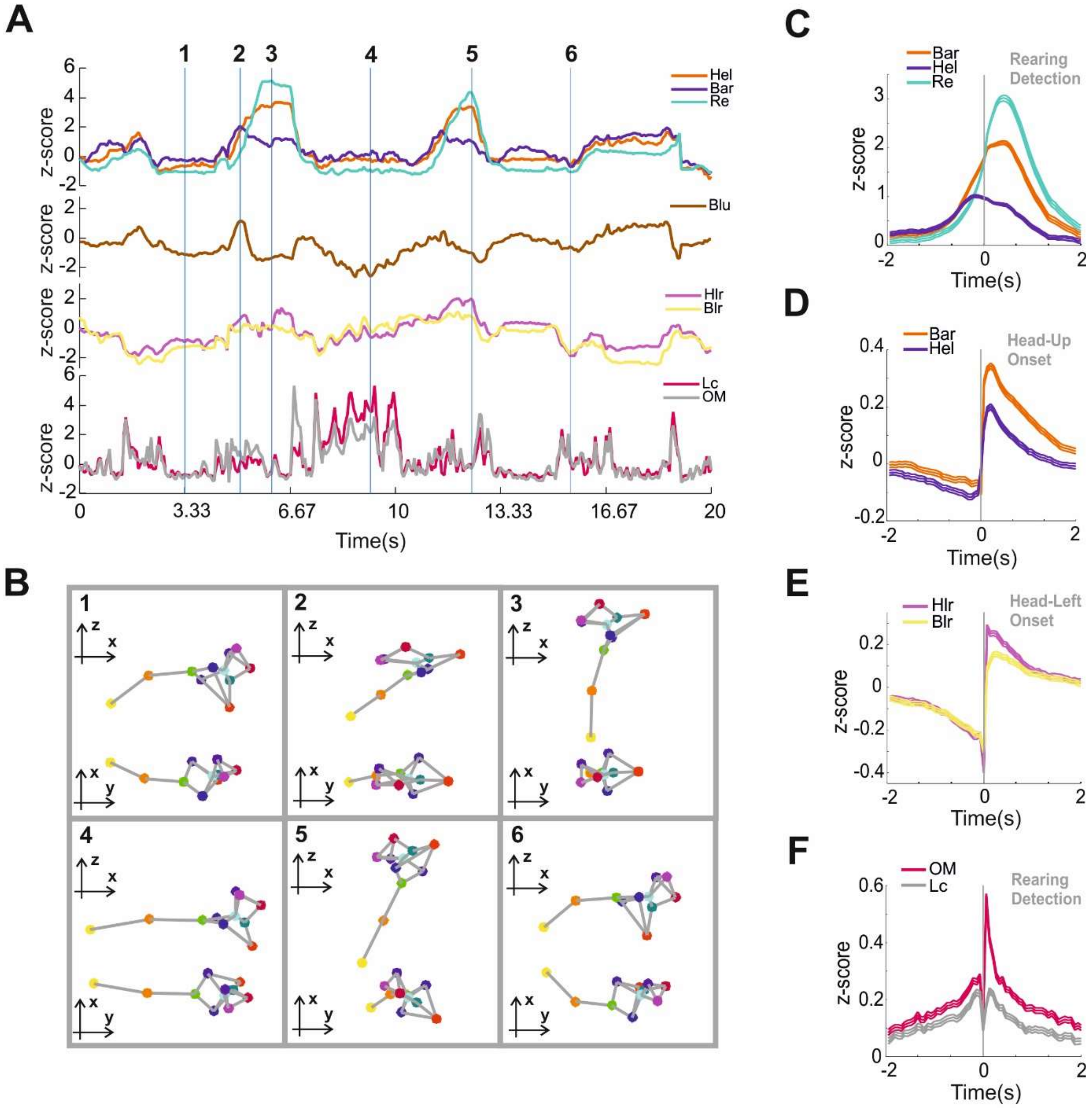

Figure S2. Supplemental analyses of behavioural state variables. Related to Figure 1: A) Representative time series for a selection of behavioural state variables (head elevation, Hel, body arch, Bar, rearing, Re, body lunge, Blu, head left/right, Hlr, body left/right, Blr, locomotion, Lc, overall motion, OM – the variables not shown are simply obtained as time derivative of the postures). **B)** Poses from six frames (indicated in panel A by vertical blue lines) are shown from side and top view. **C)** Average bodt arch (Bar), rearing (Re) and head elevation (Hel) at the onset of a rearing event (detected at time 0). Note that body arch and head elevation precede rearing. **D)** Average body arch (Bar) and head elevation (Hel) at the onset of an upward head movement (detected at time = 0). **E)** Same as panel D but for head left/right (Hlr) and body left/right (Blr) measured at onset of a leftward head movement. **F)** Average overall motion (OM) and locomotion (Lc) at the onset of a rearing event.

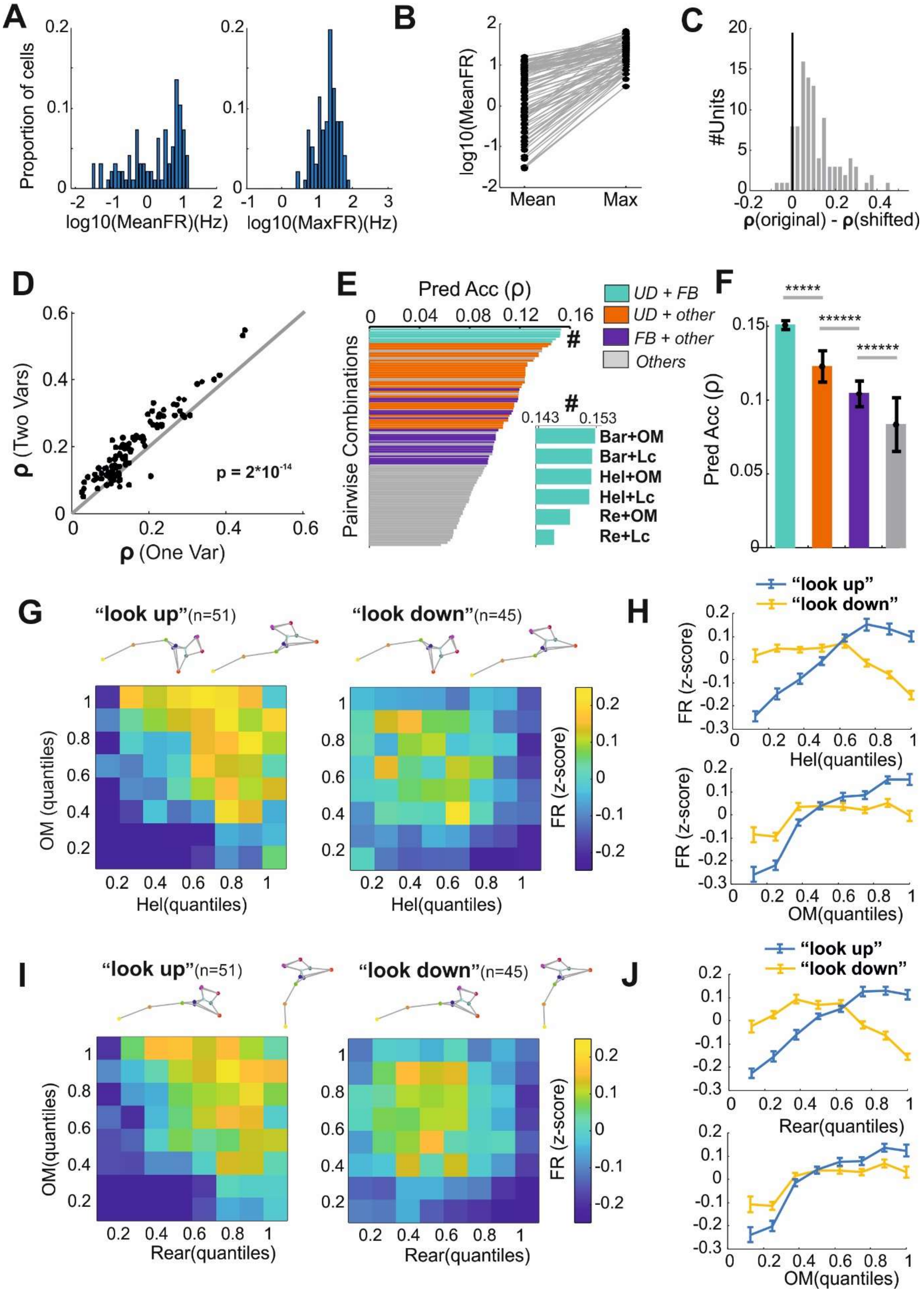
A
Proportion of cells
log10(MeanFR)(Hz)
log10(MaxFR)(Hz)
B
log10(MeanFR)
Mean
Max
C
#Units
ρ(original) - ρ(shifted)
D
ρ (Two Vars)
ρ (One Var)
p = 2*10^-14
E
Pred Acc (ρ)
Pairwise Combinations
#
0.143
0.153
Bar+OM
Bar+Lc
Hel+OM
Hel+Lc
Re+OM
Re+Lc
UD + FB
UD + other
FB + other
Others
F
Pred Acc (ρ)
*****
******
******
G
"look up"(n=51)
"look down"(n=45)
OM (quantiles)
Hel(quantiles)
FR (z-score)
H
"look up"
"look down"
FR (z-score)
Hel(quantiles)
OM(quantiles)
I
"look up"(n=51)
"look down"(n=45)
OM(quantiles)
Rear(quantiles)
FR (z-score)
J
"look up"
"look down"
Rear(quantiles)
OM(quantiles)

Figure S3. Supplemental analyses of coupling between behavioural state variables and neuronal activity in the dark. Related to Figure 2, Figure 3 and Figure 4: A) Distribution of mean (left panel) and peak firing rates (right panel) for our dataset (n = 96 units from 11 mice). **B)** Relation between mean and peak firing rates for the same dataset shown in panel A. **C)** Paired difference in prediction accuracy (measured as Person's ρ) between original and shifted predictors (n = 96 units for 11 mice). **D)** Comparison between prediction accuracy obtained with one variable (x-axis) and two variables (y-axis). **E)** Each horizontal bars indicates the average prediction accuracy for pairwise combinations of behavioural state variables (n = 96 units from 11 mice). Bars are colour-coded according to the type of variables (e.g. green for pairwise combinations of up/down postures and full body movements, see legend). The inset (indicated by #) magnifies the top six pairwise combinations. **F**) Prediction accuracy (Mean±SEM, n = 96 units from 11 mice) for each class of predictors. **G**) Z-score transformed firing rate (color coded) as function of head elevation (Hel) and overall motion (OM). The units are divided into "look-up" and "look-down" units. The two poses at the top of each panel represent the extreme upper and lower quantile for head elevation. **H**) Z-score transformed firing rates (mean±SEM, n = 52, 45, respectively look-up and look-down units) as function of head elevation (Hel, top panel) and overall motions (OM, bottom panel). **I-J)** Same as panels G-H but for rearing (Re) instead of head elevation.

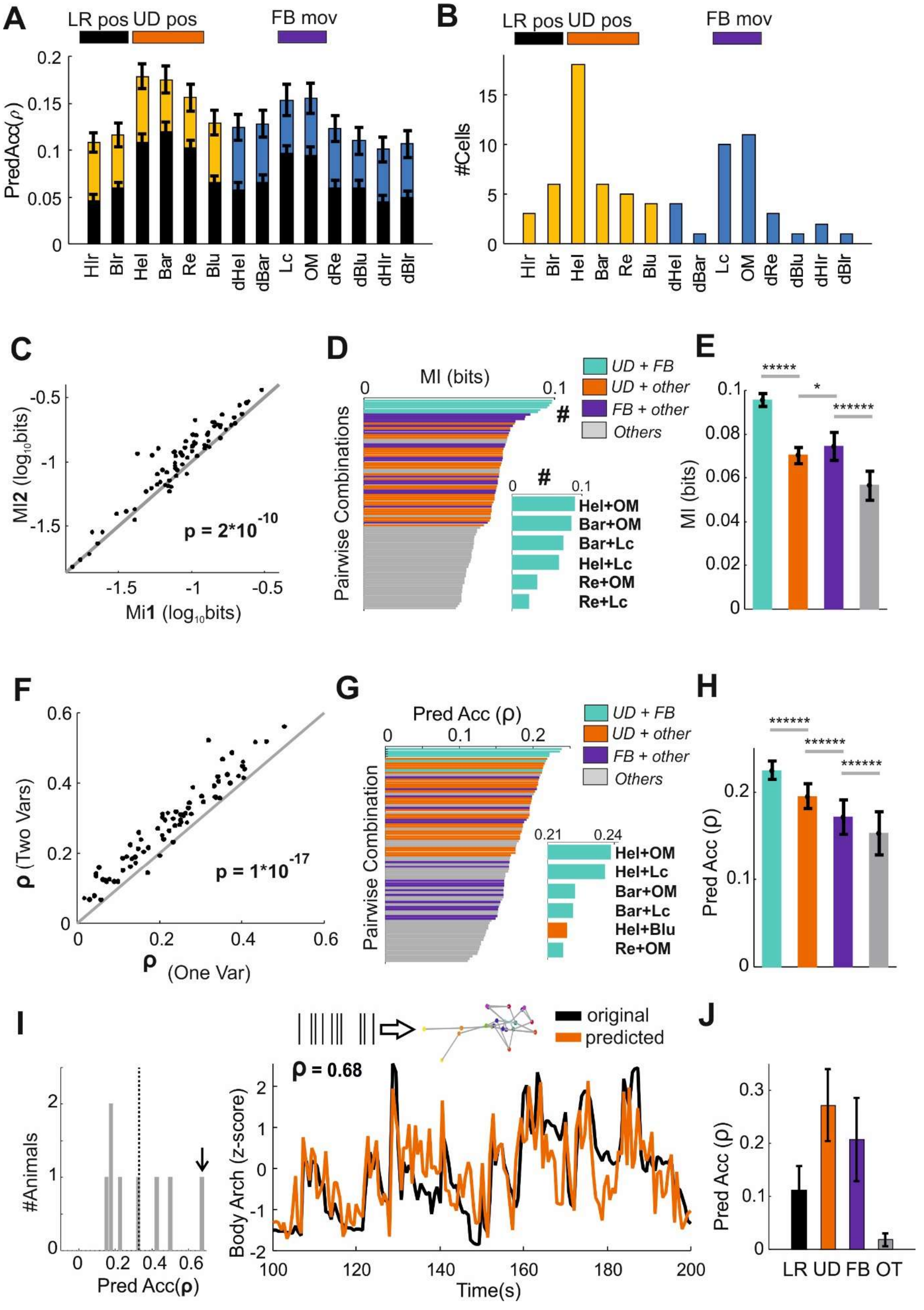

A
LR pos UD pos
FB mov
PredAcc(ρ)
Hlr Blr Hel Bar Re Blu dHel dBar Lc OM dRe dBlu dHlr dBlr
B
#Cells
C
MI2 (log10bits)
Mi1 (log10bits)
p = 2*10^-10
D
MI (bits)
Pairwise Combinations
UD + FB
UD + other
FB + other
Others
#
Hel+OM
Bar+OM
Bar+Lc
Hel+Lc
Re+OM
Re+Lc
E
MI (bits)
F
ρ (Two Vars)
ρ (One Var)
p = 1*10^-17
G
Pred Acc (ρ)
Pairwise Combination
Hel+OM
Hel+Lc
Bar+OM
Bar+Lc
Hel+Blu
Re+OM
H
Pred Acc (ρ)
I
#Animals
Pred Acc(ρ)
original
predicted
ρ = 0.68
Body Arch (z-score)
Time(s)
J
Pred Acc (ρ)
LR UD FB OT

Figure S4. Supplemental analyses of coupling between behavioural state variables and neuronal activity under photopic light. Related to Figure 5: A) Prediction accuracy (mean±SEM, n = 75 units from 8 mice) for each behavioural state variable in dark and illuminated environments (indicated respectively by black and coloured bars). **B)** Number of units best associated with each behavioural state variable according to our prediction analysis (full body movements, FB, upward/downward facing postures, UD, and left/right postures, LR, are highlighted by colour rectangles at the top). **C)** Comparison between Mutual Information (MI) conveyed by one predictor (x-axis; MI calculated as shuffle control) and two predictors (y-axis). **D)** Each horizontal bar indicates the average MI between firing rates (n = 75 units from 8 mice) and a pairwise combination of behavioural state variables. Bars are colour-coded according to the type of variables (e.g. green for up/down postures and full body movements, UD+FB, see legend). The inset (indicated by #) magnifies the top six pairwise combinations. **E**) Mutual Information (Mean±SEM, n = 75 units from 8 mice) for each class of predictors. **F-H)** Same as panels C-E but here we calculated prediction accuracy instead of Mutual Information. **I)** Distribution of prediction accuracy for all mice (n = 11; average accuracy shown as vertical dashed line). The arrow indicates an animal whose prediction based on body arch (Bar) is shown on the right panel (black = original firing rate; orange = predicted rate). **J**) Average prediction accuracy (Mean±SEM) for all animals (n=8) as function of left/right postures (LR), up/down postures (UD), full body movements (FB) and other variables (OT, which indicates the remaining 7 variables).

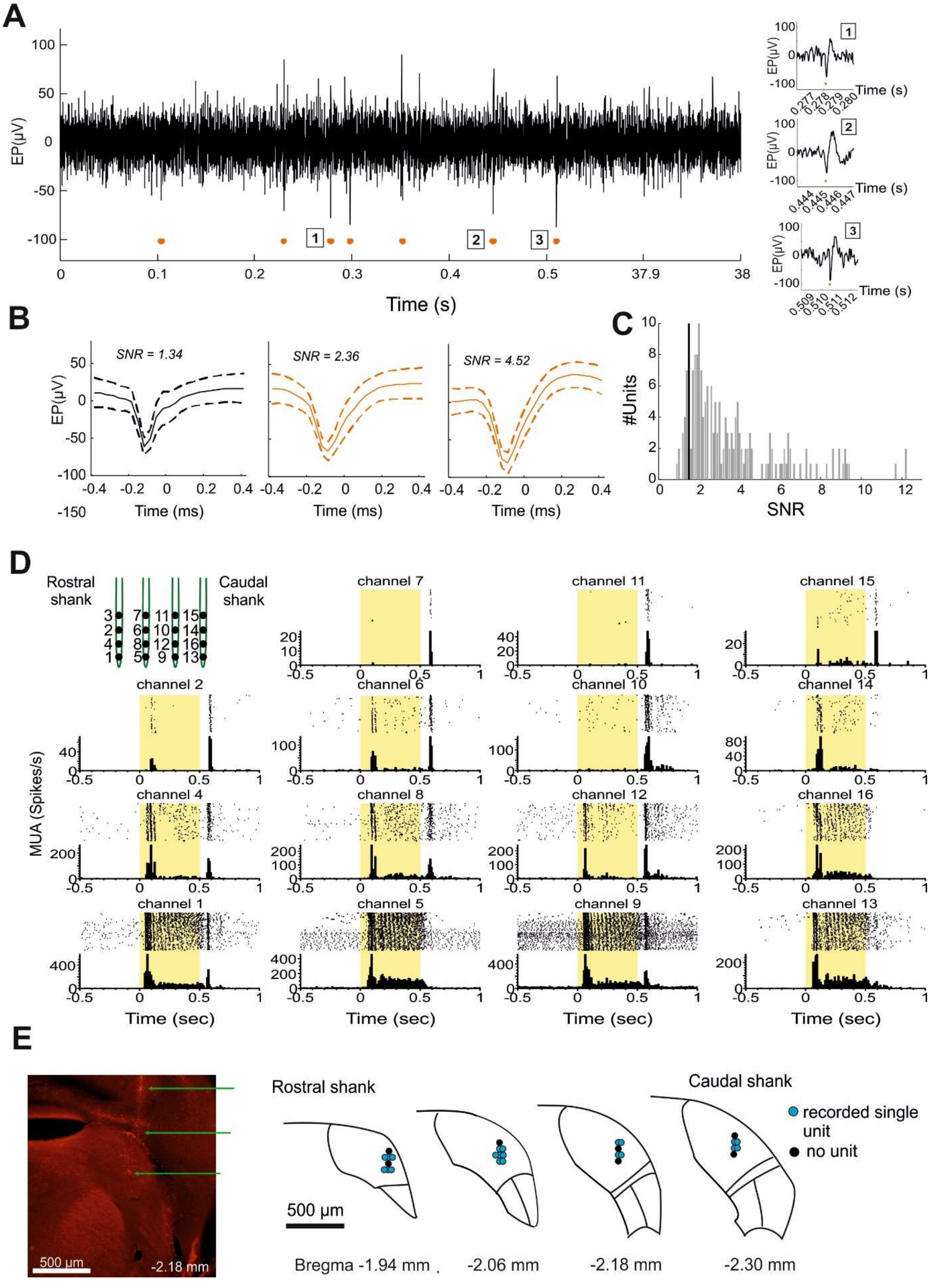
A
EP(μV)
Time (s)
B
SNR = 1.34
SNR = 2.36
SNR = 4.52
Time (ms)
C
#Units
SNR
D
Rostral shank
Caudal shank
channel 7
channel 11
channel 15
channel 2
channel 6
channel 10
channel 14
channel 4
channel 8
channel 12
channel 16
channel 1
channel 5
channel 9
channel 13
MUA (Spikes/s)
Time (sec)
E
Rostral shank
Caudal shank
recorded single unit
no unit
500 μm
-2.18 mm
Bregma -1.94 mm
-2.06 mm
-2.18 mm
-2.30 mm

Supplementary Figure 5. Extracellular recordings and anatomical localization. Related to Figure 1 and STAR Methods: A) Representative extracellular recording. Spikes detected are indicated by orange dots and three of them magnified for visual inspection (see insets 1-3). **B)** Representative sample of recorded units. Signal-to-Noise ratio (SNR) is reported at the top of each panel. Only units with SNR>1.5 were used for further analyses (here we excluded the first unit on the left since SNR=1.34). **C)** Distribution of SNR across our dataset (black vertical line indicates the threshold for inclusion). **D)** Visual responses from extracellular recordings at the end of surgical implantation of the recording electrodes. Yellow shading indicates the epoch in which full-field light stimulation was on (light onset at time 0, stimulus duration = 0.5s). Top-left panel shows the multichannel electrode layout. **E)** Histological verification of electrode placement in which fluorescent trace indicate the position of the rostral shank (left panel). Right panel shows the estimated position of isolated single units (blue dots) along rostro-caudal axis.
