# Supplementary material for: Look-up and look-down neurons in the mouse visual thalamus during freely moving exploration
Source: Curr Biol. 2022 Sep 26;32(18):3987–3999.e4. doi: 10.1016/j.cub.2022.07.049 (PMC9616738; doi:10.1016/j.cub.2022.07.049)
Supplement: Document S2. Article plus supplemental information [file mmc4.pdf]

Article

Current Biology

Look-up and look-down neurons in the mouse visual thalamus during freely moving exploration

Highlights

- Upward- and downward-facing postures are coupled to firing rate in thalamic dLGN
- The coupling to up/down postures is largely independent from locomotion
- The coupling is diverse and expressed via look-up and look-down neurons
- Look-up and look-down neuron types are stable across dark and photopic light

Authors

Patrycja Orlowska-Feuer, Aghileh S. Ebrahimi, Antonio G. Zippo, Rasmus S. Petersen, Robert J. Lucas, Riccardo Storchi

Correspondence

riccardo.storchi@manchester.ac.uk

In brief

Orlowska-Feuer et al. found that in freely moving mice, upward- and downward-facing postures are associated with excitation of two distinct populations in thalamic dLGN, defined as look-up and look-down neurons. This effect is largely independent from locomotion, is present in the dark, and is amplified under photopic light.

Orlowska-Feuer et al., 2022, Current Biology *32*, 3987–3999
September 26, 2022 © 2022 The Author(s). Published by Elsevier Inc.
https://doi.org/10.1016/j.cub.2022.07.049

CellPress

Current Biology

CellPress
OPEN ACCESS

Article

Look-up and look-down neurons in the mouse visual thalamus during freely moving exploration

Patrycja Orlowska-Feuer,[1] Aghileh S. Ebrahimi,[1] Antonio G. Zippo,[2] Rasmus S. Petersen,[1] Robert J. Lucas,[1] and Riccardo Storchi[1,3,4,*]
[1]University of Manchester, Faculty of Biology, Medicine and Health, School of Biological Science, Division of Neuroscience and Experimental Psychology, Oxford Road, M139PL Manchester, UK
[2]Institute of Neuroscience, Consiglio Nazionale delle Ricerche, Via Raoul Follereau, 3, 20854 Vedano al Lambro, Italy
[3]Twitter: @riccardostorch1
[4]Lead contact
*Correspondence: riccardo.storchi@manchester.ac.uk
https://doi.org/10.1016/j.cub.2022.07.049

SUMMARY

Visual information reaches cortex via the thalamic dorsal lateral geniculate nucleus (dLGN). dLGN activity is modulated by global sleep/wake states and arousal, indicating that it is not simply a passive relay station. However, its potential for more specific visuomotor integration is largely unexplored. We addressed this question by developing robust 3D video reconstruction of mouse head and body during spontaneous exploration paired with simultaneous neuronal recordings from dLGN. Unbiased evaluation of a wide range of postures and movements revealed a widespread coupling between neuronal activity and few behavioral parameters. In particular, postures associated with the animal looking up/down correlated with activity in >50% neurons, and the extent of this effect was comparable with that induced by full-body movements (typically locomotion). By contrast, thalamic activity was minimally correlated with other postures or movements (e.g., left/right head and body torsions). Importantly, up/down postures and full-body movements were largely independent and jointly coupled to neuronal activity. Thus, although most units were excited during full-body movements, some expressed highest firing when the animal was looking up (“look-up” neurons), whereas others expressed highest firing when the animal was looking down (“look-down” neurons). These results were observed in the dark, thus representing a genuine behavioral modulation, and were amplified in a lit arena. Our results demonstrate that the primary visual thalamus, beyond global modulations by sleep/awake states, is potentially involved in specific visuomotor integration and reveal two distinct couplings between up/down postures and neuronal activity.

INTRODUCTION

A key role of vision is to guide motor actions.[1–4] In turn, motor actions modify the visual experience through self-motion and changes in head and body postures so that appropriate interpretation of incoming visual information depends on these parameters.[5–7] For example, mice respond with freezing to a sweeping object flying overhead[2] but with pursuit hunting to a similar object moving at ground level,[3] suggesting that selection of the appropriate action requires integration of head and body positioning with the visual input. This process of integration has traditionally been studied at high levels of the hierarchical visual pathway (e.g., posterior parietal cortex[8,9]; rodent lateral posterior thalamus and primate pulvinar[10,11]). Spontaneous and visually evoked activity in primary visual cortex has also been shown to be strongly affected by locomotion in head-fixed preparations[12–15] and head rotations along different axes in freely moving animals.[16,17]

The role of primary visual thalamus in integrating the visual input with postures and movements is still unresolved. The traditional view of primary visual thalamus is that of a relay station between the retina and cortex. However, retinal input only represents a small fraction of the afferent synapses[18,19] and other brain projections, arising from brainstem, visual cortex, and thalamic reticular nucleus (TRN), can influence the flow of visual information.[18,20,21] A vast body of work has shown that spontaneous and evoked patterns of firing activity (e.g., tonic or bursting) and the gain of visual responses are determined according to anesthesia, sleep, and awake states (see e.g., Hubel,[22] Jeczmien-Lazur et al.,[23] Saalmann and Kastner,[24] Storchi et al.,[25] and Steriade et al.[26]). It has also been shown that neurons in visual thalamus (and even in the retina[27,28]) are modulated by locomotion in a head-fixed preparation.[29,30] However, since in this preparation locomotion is strongly associated with arousal,[27–29] it is not clear whether such modulation reflects a generalized state of alertness or a specific effect of locomotion. Most importantly, a description of which postures and movements modulate neuronal activity in natural unconstrained conditions is still missing. The primary goal of this study was to

This is an open access article under the CC BY license (http://creativecommons.org/licenses/by/4.0/).

address this deficit by measuring simultaneously postures, movements, and firing activity in the primary visual thalamus in freely moving mice.

Success of this endeavor is dependent upon a method to accurately quantify the wide repertoire of postures and movements available to freely moving mice. Computational methods to track body parts in 3D and use these to reconstruct pose at frame-by-frame resolution are increasingly available.[31–34] We have previously developed such an approach suitable for mice,[34] and here, we extend it to measure a wide variety of 3D movements and postures. We find that the higher dimensional description of behavior enabled by this approach is key to understanding motor influences on the early visual system. Thus, although we confirm that high levels of motor activity (including but not limited to the locomotion available to head-fixed animals) excites the primary visual thalamus, we find that head and body postures are at least as influential in defining neuronal activity in this region. Moreover, although movement provides a general increase in firing, the impact of postures is more specific, with a subset of neurons excited by poses characterized by looking upward (''look-up'' neurons), whereas a different set are excited by poses associated with looking downward (''look-down'' neurons). Our discovery that electrophysiological activity in the primary visual thalamus is influenced by posture during natural exploration raises the possibility that thalamic processing of visual information can be flexibly modulated according to specific visuomotor behaviors.

RESULTS

Spontaneous exploration in freely moving animals is defined by independent sets of postures and movements

Mice were recorded by using four synchronized cameras (Figure S1A) during spontaneous exploration of an open field arena either in the dark or under steady illumination (respectively n = 11, 8 animals). To capture posture and movements in freely moving mice, we first performed 3D reconstruction of head and body in animals implanted with multichannel microelectrodes (STAR Methods) in dorsal lateral geniculate nucleus (dLGN). Eleven landmarks were identified on the mouse's body and microelectrode head stage and were used for tracking the animals (Figure 1A). The 2D tracking data were then triangulated to generate an initial 3D reconstruction of the animal (STAR Methods). Outliers and missing data in the 3D reconstruction were then corrected by extending a previously developed approach[34] (STAR Methods). The corrected 3D data provided a robust estimate of the animal body over time (Video S1).

From the 3D reconstruction, we quantified the postures and movements of the mouse in terms of fourteen behavioral variables. Hereafter, we will define this set of variables as the behavioral state of the animal. Head postures were quantified by head elevation and left/right angles (Figure 1B; Video S2; Hel, head elevation; Hlr, head left/right). Full-body postures were quantified by projection on the first 3 eigenposes (Figure 1B; Video S2; Bar, body arch; Blr, body left/right bend; and Blu, body lunge), sufficient to explain 80% of changes in the body shape, and by rearing (Figure 1B; Video S2; Re, rearing). Six movements were quantified by the temporal derivative of the above postures (dHel, dHlr, dBar, dBlr, dBlu, and dRe). Locomotion was quantified by speed of movement on the x-y plane of the arena (Figure 1B; Video S2; Lc, locomotion). Finally, we quantified overall motion (Figure1B; OM, overall motion) by measuring the 3D Euclidean distance between all body points in consecutive frames.

Given biomechanical constraints, we expect some postures and movements to correlate with each other. Therefore, we set out to identify relevant groupings among behavioral state variables. To do that, we first applied mutual information (MI) to quantify linear and nonlinear correlations between all pairs. This analysis revealed two main groups that corresponded to postures and movements, respectively (Figure 1C). The existence of these two groups was confirmed by a hierarchical clustering analysis (Figure 1D, HIGH hierarchical level partition). Hierarchical clustering also revealed that at a finer grain of analysis, there were three prominent sub-groups (Figure 1D, LOW hierarchical level partition). (1) Head elevation, body arch, and rearing were all associated with animal looking up or down, and therefore, we defined this group as upward/downward facing postures (Figure 1D, UD). (2) Left/right head turn and left/right body bend, which we defined altogether as left/right postures (Figure 1D, LR). (3) Overall motion and locomotion defined hereafter as full-body movements (Figure 1D, FB). The same sub-groups were observed both in the dark and under ambient illumination (Figures S1B–S1E), indicating that these sub-groups represent a stable and robust feature of mouse behavior. Other sub-groups were also identified (e.g., dHlr and dBlr; Figure 1D); however, these were not consistent across dark and ambient illuminations (Figures S1B–S1E).

Visual inspection indicated that individual variables within each subgroup, although correlated, captured distinct components of the mouse behavior (see representative times series in Figures S2A and S2B). Thus, body arch and head elevation typically preceded rearing (Figure S2C). Changes in head posture, captured by head elevation and head left/right turns, could also occur with reduced or minor changes in full-body posture, captured by body arch and body left/right bend (Figures S2D and S2E). Overall motion, although strongly associated with locomotion, also encompassed vertical actions like rearing (Figure S2F).

Upward-/downward-facing postures and full-body movements dominate firing-rate modulation in visual thalamus in the dark

We set out to determine the extent to which behavioral state variables involved in spontaneous exploratory behavior were coupled to neuronal activity. In order to eliminate the possibility that such couplings could arise from associations between behavior and visual experience, we first ran these recordings in the dark (see Figures S3A and S3B for mean and peak firing rates for single units in this dataset).

From simple visual inspection, we observed that some units exhibited an increase or decrease in firing that accompanied step changes in behavioral state variables (Figures 2A and 2B). Joint visualization of behavioral time series and spike patterns also suggested an association between behavioral variables and spiking activity (Figures 2C and 2D).

To quantify these effects, we performed a cross-correlation analysis between individual variables and single-unit firing rates

CellPress
OPEN ACCESS

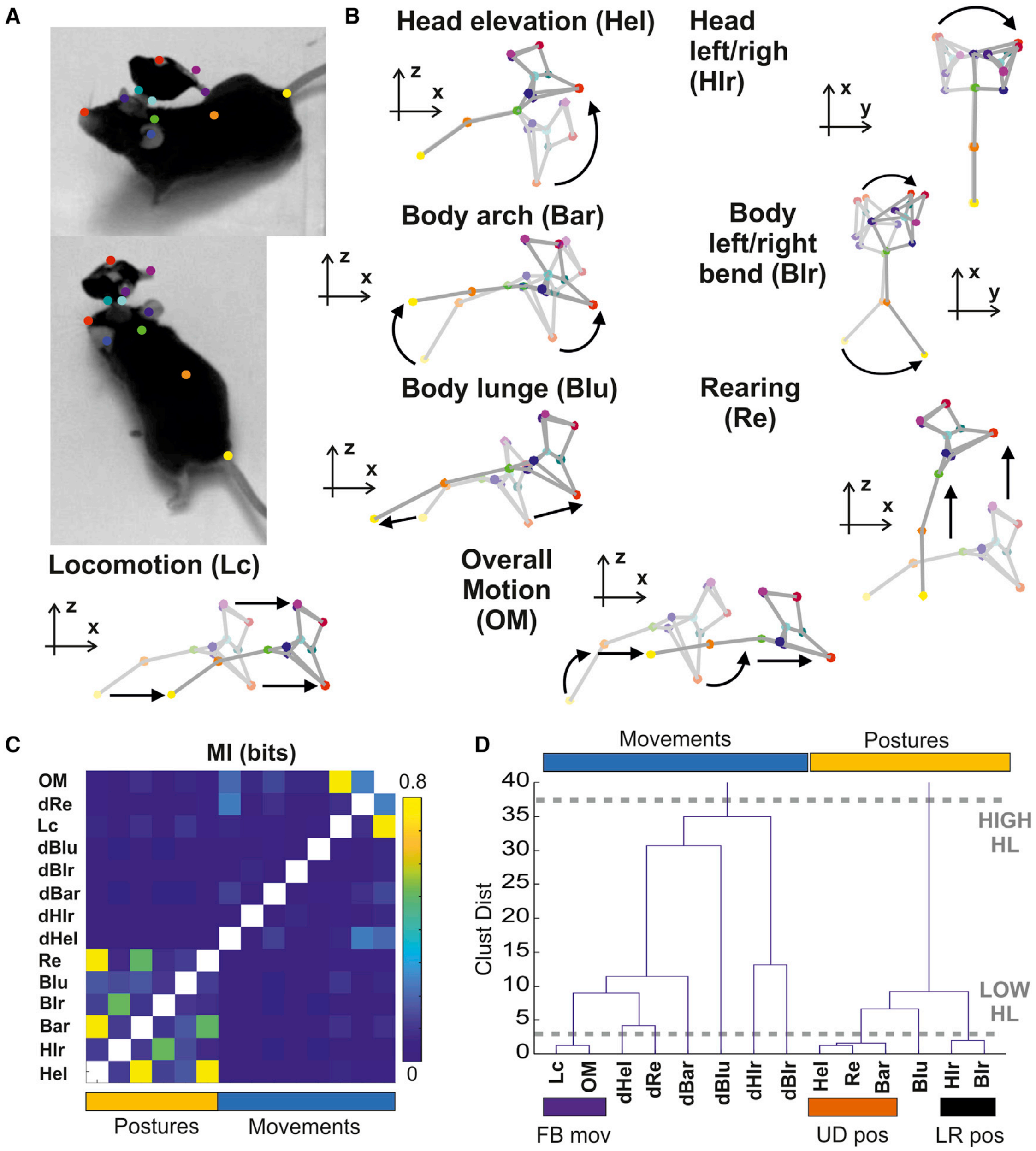

Figure 1. Three-dimensional reconstruction and classification of postures and movements

(A) Visualization of the eleven body landmarks applied to the animal body and the recording head stage.

(B) Representation of a selection of 8 behavioral state variables. Each vignette illustrates changes in body posture and position according to an individual behavioral state variable. The arrows always point toward the highest positive values. The axes indicate the view point (x-z for side views and x-y for top views). The other behavioral state variables not represented here (dHel, dHlr, dBar, dBlr, dBlu, and dRe) are obtained as temporal derivatives of the associated postures.

(C) Matrix of pairwise mutual information between behavioral state variables. Postures and movements are indicated by cyan and orange bars.

(D) Hierarchical clustering for all behavioral state variables. Two levels of granularities are highlighted (dashed gray lines) indicating, respectively, a high hierarchy (HIGH hierarchical level: postures and movements, marked by cyan and yellow bars on top of the graph) and a low hierarchy (LOW hierarchical level: full-body movements, up/down postures, left/right postures, marked by respectively by purple, orange, and black bars).

See also Figures S1 and S2 and Videos S1 and S2.

that revealed that a large fraction of units exhibited significant cross-correlation peaks (Figure 2E; shuffle test; STAR Methods). The most common correlations were with variables encompassing up/down postures and full-body movements (Figure 2E, UD and FB). For most variables, a comparable number of units exhibited either positive or negative correlation peaks, whereas for full-body movements, units exhibited almost exclusively positive correlations (Figure 2E). Across all variables, the correlation peaks occurred on average around time zero (see Figure 2F), indicating that this aspect of neuronal activity neither

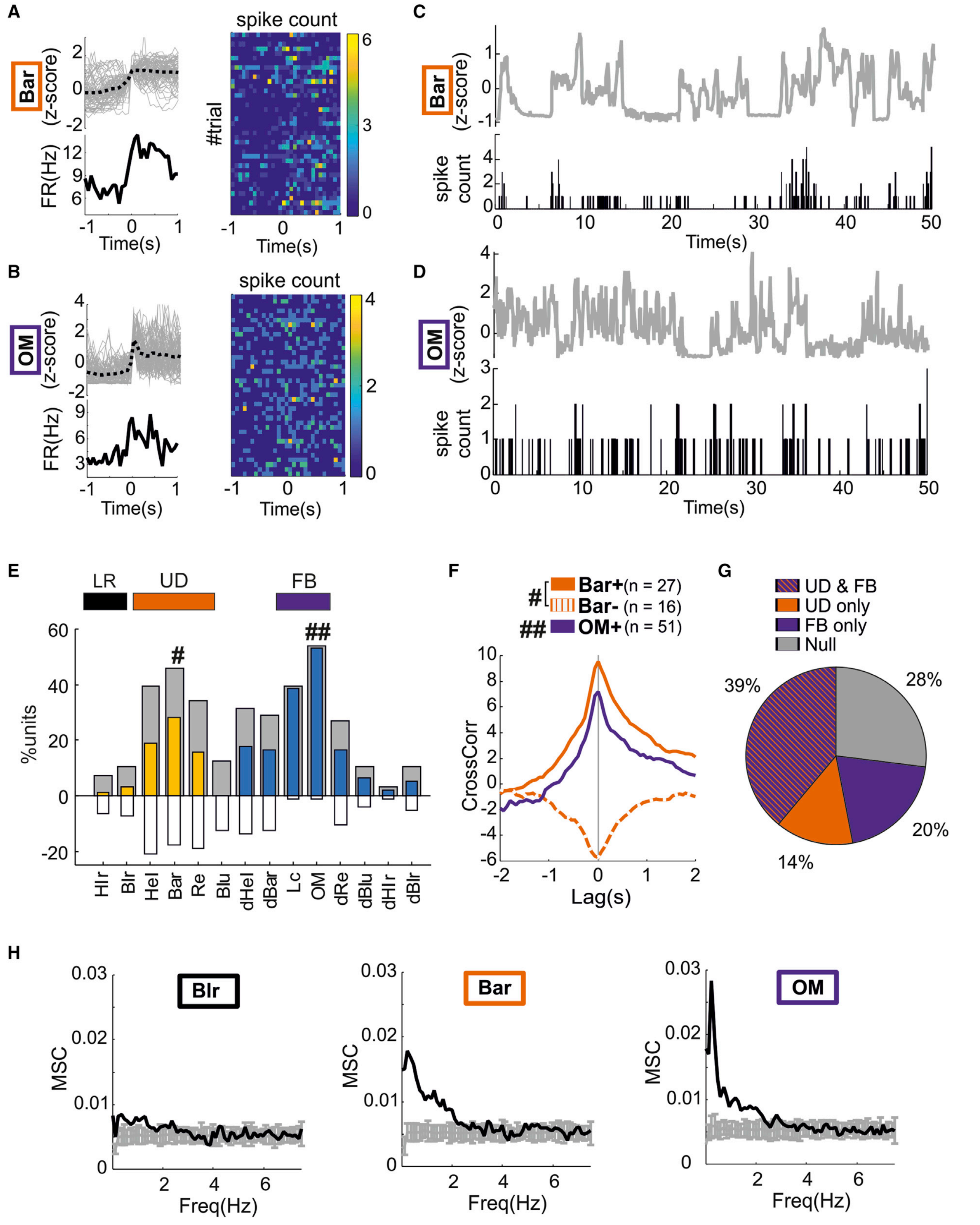

Figure 2. Upward-/downward-facing postures and full-body movements dominate firing rate modulation in visual thalamus in the dark

(A) Step-increases in body arch (bar, top-left panel, gray lines indicate individual events, and dotted black line indicates the average increase), average change in averaged firing rate (bottom-left panel) and trial-bin count showing spike patterns for individual events (right panels, bin duration = 66 ms).

(legend continued on next page)

systematically predicted nor lagged actions but rather provided a near instantaneous reflection of the behavioral state. Overall, >70% units (n = 69/96 units from 11 mice) were linearly correlated with up/down postures and/or full-body movements (Figure 2G), and the remaining units had no significant correlations with any other variable. A coherence analysis further revealed that the linear coupling between behavioral state variables and single-unit activity was concentrated in the 0–2 Hz range (Figure 2H).

In order to capture both linear and nonlinear correlations between behavioral state variables and neuronal activity, we performed three types of additional analyses: first, we calculated MI between single variables and firing activity of individual units (Figures 3A and 3B); then, we performed cross-validated predictions of single-unit firing from individual variables (Figures 3A, 3C, and 3D), and finally, we applied the same predictive approach to estimate single behavioral state variables from the firing rates of all the units that we recorded simultaneously from individual animals (Figures 3E and 3F).

First, for each unit, we identified the single behavioral state variable with highest MI (corresponding to that unit's "favored" behavioral variable). Most units (n = 80/96 units from 11 mice) favored variable was either with an up/down posture or a full-body movement (Figure 3A, UD and FB, solid color bars). Among up/down postures, units conveyed higher information for body arch than for rearing (p = 0.0056, sign = 62, n = 96 units from 11 mice, sign-test), whereas body arch and head elevation were not significantly different (p = 0.1253, sign = 40, n = 96, sign-test). Among full-body movements, information conveyed by locomotion and overall motion was also comparable (p = 0.36, sign = 53, n = 96 units from 11 mice, sign-test). Across the recorded neuronal population, the amount of information was proportional to the firing rate, so that units with higher firing rates carried higher information (Figure 3B, p < 0.0001, n = 96 units from 11 mice, t test). These results indicate that the modulation of dLGN neurons by posture mainly reflected sensitivity to body arch, which encompasses head elevation, whereas modulation by movements was dominated by locomotion.

We then trained a model (based on XGBoost[35,36]; STAR Methods) to predict firing rate from individual behavioral variables. We evaluated prediction accuracy by calculating Pearson's linear correlation between recorded and predicted spike counts on cross-validated data. Based on these results, for each unit, we identified the best predictor, i.e., the variable associated with highest Pearson's correlation. Across our dataset, the best predictors captured significant amounts of variability (ρ = 0.16 ± 0.08, mean ± SD; Figure 3C). To exclude the possibility that this result was an artifact of our analyses, we shifted the behavioral variables by half a recording epoch to break their associations with neuronal activity while preserving their temporal structure (STAR Methods). Prediction accuracy dropped indicating that our analyses capture genuine coupling between behavioral variables and neuronal activity (Figure S3C). Consistently with MI analyses, up/down postures and full-body movements were the best predictors for 76% of the units (Figure 3A, striped bars) substantially outperforming left/right postures and all other variables (Figure 3D, p < 0.0005 across all comparisons, sign-test, n = 96 from 11 animals). Prediction accuracy was also proportional to the units' firing rate (p < 0.0001, n = 96 from 11 animals, t test).

Finally, we used the same XGB models to predict behavioral state variables from population activity (i.e., the spike counts from all units that were simultaneously recorded from each animal). We found that up/down postures and full-body movements were the best predicted variables, providing substantial increases in accuracy over left/right postures and other variables (Figures 3E and 3F, p = 0.0002, χ^2 = 19.22, n = 11 mice, Kruskal-Wallis test).

Overall, these results show that during spontaneous exploration in the dark, neurons in visual thalamus are modulated by behavioral state, and the effect is associated with upward-/downward-facing postures and full-body movements.

Single-unit firing is jointly modulated by upward-/downward-facing postures and full-body movements in the dark

Do individual units encode single behavioral variables and do they encode more than one? To answer this, we first estimated MI between single-unit activity and all possible pairs of variables. For each unit, we then selected the pair associated with the highest information. We first asked whether pairs of variables provided more information than individual ones. To provide the fairest comparison, we re-estimated MI for individual variables by using pairs in which the values from one variable were shuffled over time to remove its association with neuronal activity (STAR Methods). We found that most units conveyed more

(B) Same as (A) but for a unit coupled to increases in overall motion (OM).
(C) Time series of body arch (bar) and spike counts (bin duration = 66 ms) for the same unit shown in (A).
(D) Time series of overall motion (OM) and spike counts (bin duration = 66 ms) for the same unit shown in (B).
(E) Results of the cross-correlation analyses (n = 96 units from 11 mice). Colored and white bars indicate the percentage of units with significant positive (colored) and negative (white) association with each behavioral state variable (Hlr, head left-right; Blr, body left/right; Hel, head elevation; Bar, body arch; Re, rearing; Blu, body lunge; Lc, locomotion; OM, overall motion—the other labels dHel, dBar, dRe, dBlu, dHlr, and dBlr represent the temporal derivatives of Hel, Bar, Re, Blu, Hlr, and Blr). Gray bars indicate the overall percentage of units with a significant association. Full-body movements, up/down and left/right postures are highlighted by horizontal-colored rectangles at the top. Hash symbols indicate the two most prevalent variables for postures and movements (# and ##, respectively).
(F) Average cross-correlation across all significant units for the two most prevalent variables (Bar indicted by #, OM indicated by ##; Bar+ and Bar−, respectively, indicate units with positive and negative peak correlations).
(G) Percentage of units with a significant cross-correlation peak for both up/down postures (UD) and full-body movements (FB) variables (striped bars), for UD only (orange) and FB only (purple). The remaining units (gray) have no significant peaks for any of the remaining variables.
(H) Average magnitude squared coherence (MSC, black lines) across the full dataset of recorded units (n = 96 units from 11 mice) for body left/right (Blr, left panel), body arch (Bar, middle panel) and overall motion (OM, right panel). Gray bars indicate the average MSC (±2 SD) obtained by random shifts the behavioral state variables time series in respect to the spike patterns (n = 100 shifts).
See also Figure S3.

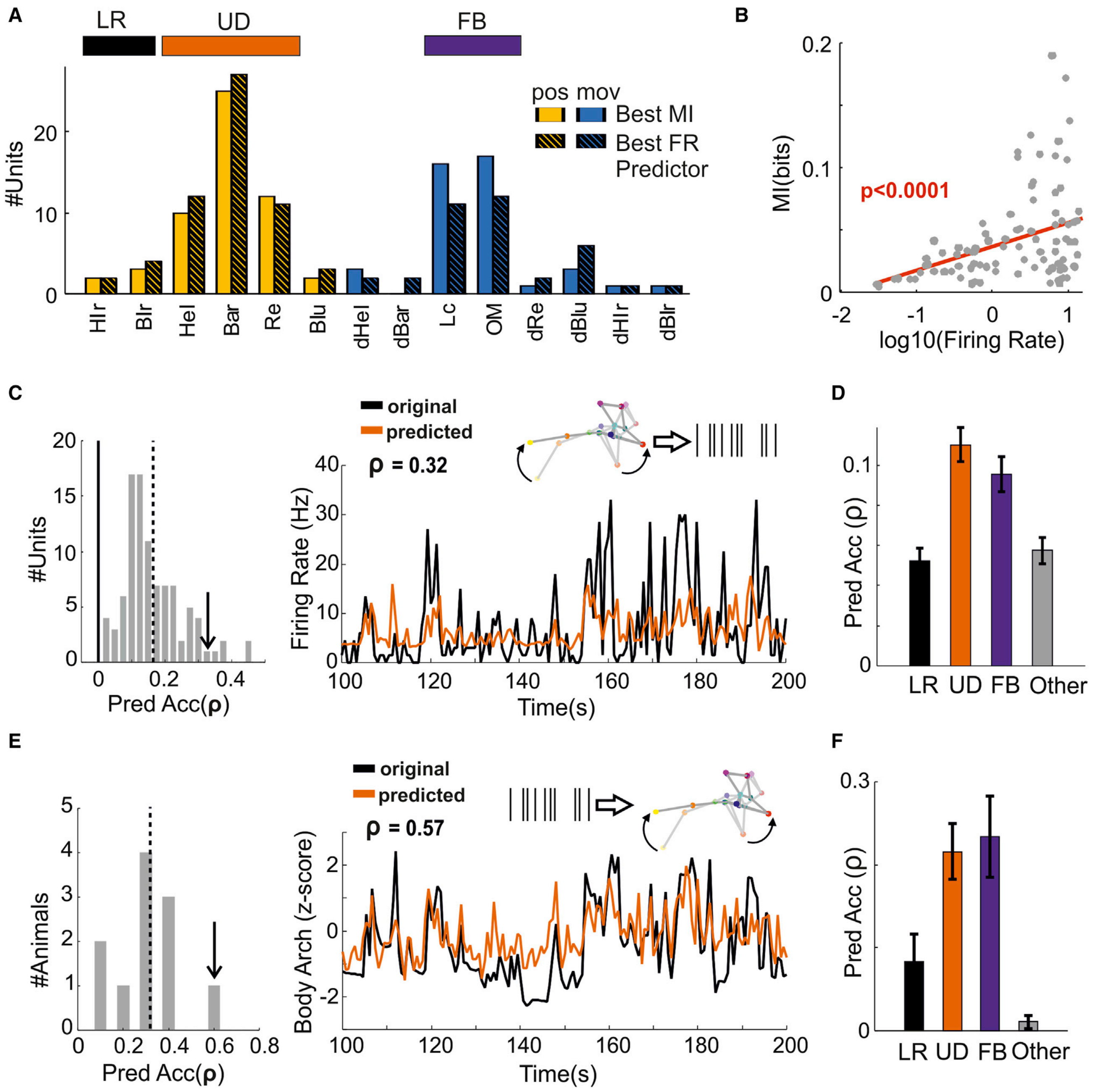

Figure 3. Upward-/downward-facing postures and full-body movements are the best predictors of neuronal activity in visual thalamus in the dark

(A) Number of units best associated with each behavioral state variable (full-body movements, [FB], upward-/downward-facing postures, [UD], and left/right postures, [LR] are highlighted by color bars at the top). Solid color bars indicate results from mutual information analyses while striped bars indicate results from prediction analyses.

(B) Mutual information as function of firing rate (in log units) across all cells (n = 96 from 11 mice).

(C) Distribution of prediction accuracy for all units recorded (left panel, n = 96 units from 11 mice; average accuracy shown as vertical dashed lines). The arrow indicates a unit whose prediction as function of body arch (Bar) is shown on the right panel (black, original firing rate; orange, predicted rate).

(D) Prediction accuracy (mean ± SEM, n = 96 units from 11 mice) for all units as function of left/right postures (LR), up/down postures (UD), full-body movements (FB), and the other 7 variables.

(E) Distribution of prediction accuracy for all mice recorded (n = 11). The arrow indicates an animal whose prediction is shown on the right panel (prediction based on n = 22 units; black, measured Bar; orange, predicted Bar).

(F) Prediction accuracy (mean ± SEM) for all mice (n = 11) as function of left/right postures (LR), up/down postures (UD), full-body movements (FB), and the other 7 variables.

See also Figure S3.

CellPress
OPEN ACCESS

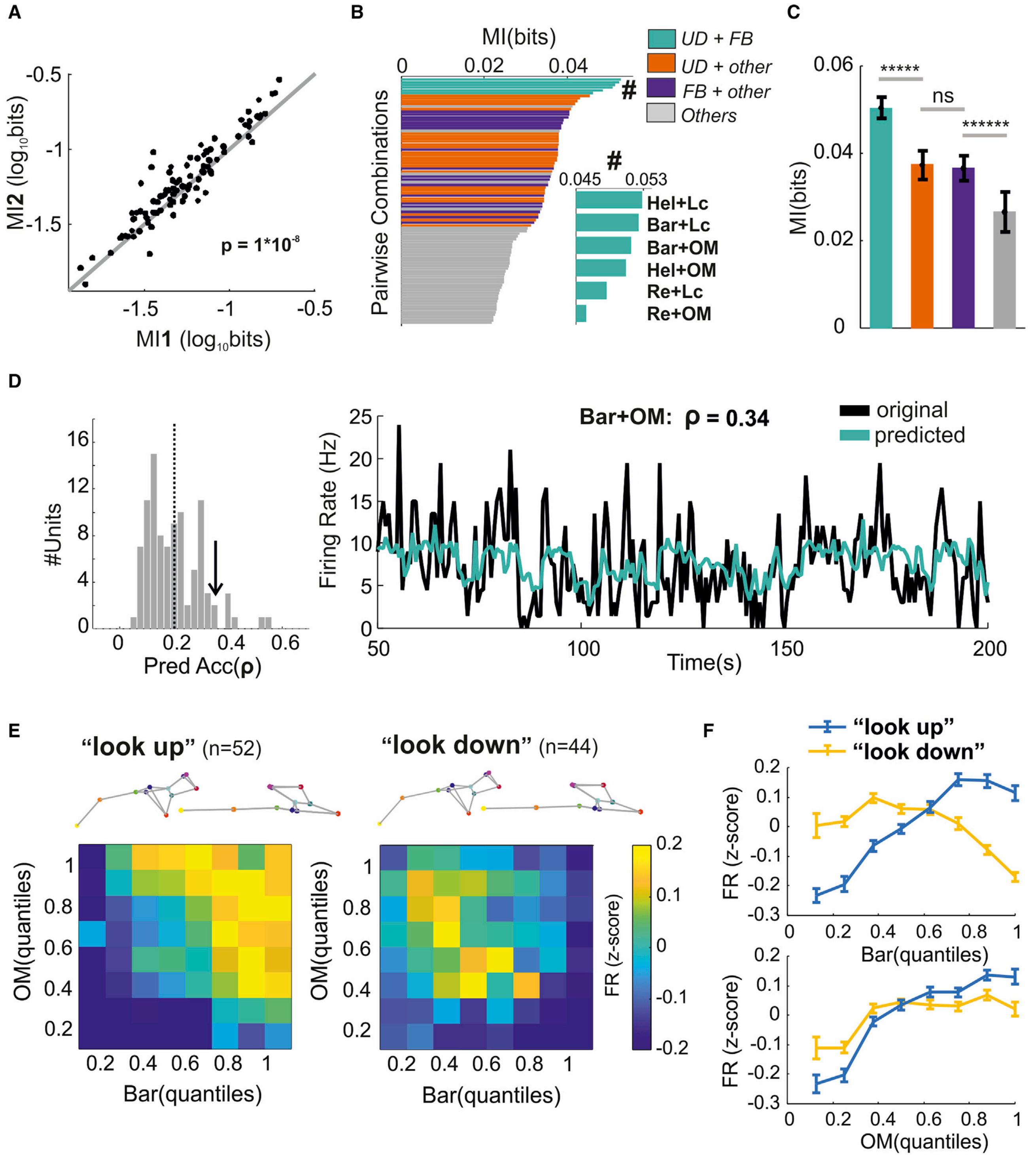

Figure 4. Single-unit firing is jointly modulated by upward-/downward-facing postures and full-body movements in the dark

(A) Comparison between mutual information (MI) conveyed by one predictor (MI1, x axis; MI1 calculated as shuffle control; STAR Methods) and two predictors (MI2, y axis).

(B) Each horizontal bars indicates the average mutual information (MI between firing rates of 96 units and a pairwise combination of behavioral state variables (n = 96 units from 11 mice). Bars are color-coded according to the type of variables (e.g., green for pairwise combinations of up/down postures and full-body movements, see the legend). The inset (indicated by #) magnifies the top six pairwise combinations.

(C) Mutual information (mean ± SEM, n = 96 units from 11 mice) for each class of predictors.

(legend continued on next page)

information about variable pairs than about individual variables (Figure 4A, $p = 1 \times 10^{-8}$, sign-test, n = 96 units from 11 mice). This was the case also when we compared average MI across animals (p = 0.01, sign-test, n = 11 animals). We next asked which variables were most strongly coupled to neuronal activity. We found that pairwise combinations of up/down postures and full-body movements conveyed the highest MI (Figure 4B), providing, on average, a 90% increase over other pairwise combinations that did not include those variables (Figure 4C).

Consistently with MI analyses, pairs of behavioral state variables provided better prediction of single-unit activity compared with single variables (Figures S3D and S3E, $p = 2 \times 10^{-14}$, sign-test, n = 96 units from 11 mice; the distribution of prediction accuracies is reported in Figure 4D). Up/down postures and full-body movements substantially outperformed other predictors (Figure S3F).

Finally, we asked which and how many different types of modulations could be observed at a single-unit level. To answer this question, we performed an unsupervised clustering analysis (based on community detection[37]; STAR Methods). Each unit was represented as a bivariate histogram of the average firing rates as function of body arch and overall motion (the two strongest predictors). The clustering process then automatically determined the number of distinct types (STAR Methods). This analysis revealed two classes of units: "look-up" units, most active when the animal assumed an upward-facing posture (Figure 4E, left panel; Figure 4F, top panel; n = 52 from 11 mice), and "look-down" units, most active when the animal assumed a downward-facing posture (Figure 4E, right panel; Figure 4F, bottom panel; n = 44). Both "look-up" and "look-down" units were positively modulated by increased levels of motor activity (Figure 4F, look-up: $p = 1 \times 10^{-37}$, 8×10^{-12}, $\chi^2 = 190.12, 66.4$; n = 52, 44; Kruskal-Wallis test for look-up and look-down units). Similar clustering results were obtained by combining overall motion with head elevation or rearing (Figures S3G–S3J).

These results show that dLGN units are jointly coupled to overall motion and up/down postures, with ~54% units most excited during upward-facing postures and the remaining fraction during downward-facing postures.

Modulation of single-unit activity by behavioral state is maintained and amplified by ambient illumination

All modulations described so far were obtained by recording the animal in the dark, i.e., in the absence of visual stimulation. We next asked whether these modulations would persist when the visual thalamus is additionally stimulated by visual inputs. To address this question, we repeated our freely moving experiments, but this time with the arena illuminated (within the photopic range; STAR Methods).

In the light arena, average values of MI across all postures and movements were higher than those in the dark (Figure 5A, $p < 0.005$ for all variables, rank-sum tests, n = 96 and 75 units from 11 and 8 mice recorded, respectively, in for dark and light conditions). All other results were qualitatively recapitulated. Up/down postures and full-body movements were still associated with largest MI values in the majority of the cells (81%, n = 61/75 units from 8 mice; Figure 5B). Most units conveyed more information about pairs of behavioral state variables than about individual variables (Figure S4C, $p = 5 \times 10^{-11}$, sign = 65, n = 75 units from 8 mice), and pairwise combinations of up/down postures and full-body movements conveyed the highest MI (Figures S4D and S4E). Results based on the predictive modeling approach were consistent with MI analysis (Figures 5C and S4F–S4J). Clustering analysis revealed two classes of units that qualitatively matched the "look-up" and "look-down" units observed in the dark (Figures 5D and 5E).

Finally, we asked whether individual neurons maintained the same tuning to behavioral state variables in darkness and in light. In order to avoid potential artifacts due to electrode movements across different days, we analyzed a dataset in which animals were recorded both in dark and light within the same experimental session (n = 58 units from 5 mice). We found that the tuning for the strongest predictors, body arch and overall movement, was significantly preserved (Figures 5F and 5G).

These results show that the coupling between behavioral variables and single-unit activity described in the dark is maintained and amplified by visual stimulation.

DISCUSSION

In everyday life, visual processing is concurrent with movements and changes in posture. It follows that to understand visual processing, we also need to understand how specific actions affect neuronal activity along the visual pathway. Previous studies found strong modulations of neuronal activity by motor activity along the visual pathway and throughout the brain.[12–15,29,30,38,39] However, since most studies were performed in head-fixed animals, it is still unclear which aspects of behavior would couple to neuronal activity in natural freely moving conditions. Our study aimed to fill this gap in the mouse dLGN, the key subcortical station linking the retina to the primary visual cortex.

Our first result was that upward- and downward-facing postures affected firing rate of >50% of neurons in dLGN. Encoding of head and body postures was previously described in the rat posterior parietal cortex and frontal motor cortex[40] but, to the best of our knowledge, not in conventional visual centers. Most of the studies on visual processing focused on the effect of locomotion on a treadmill and revealed that this behavior substantially affect neuronal activity in primary visual cortex (see e.g., Niell and Stryker,[12] Saleem et al.,[13] Keller et al.,[14] Bennett et al.,[15] and Vinck et al.[41]), and dLGN and LP regions of the visual thalamus.[29,30,42] Since those studies were performed in

(D) Distribution of prediction accuracy for all units recorded (left panel, n = 96 from 11 mice; average accuracy shown as vertical dashed line). The arrow indicates a unit whose prediction based on body arch (Bar) and overall motion (OM) is shown on the right panel (black, original firing rate; green, predicted rate).
(E) *Z* score transformed firing rate (color coded) as function of body arch (Bar) and overall motion (OM). The units are divided into "look-up" and "look-down" units. The two poses at the top of each panel represent the extreme upper and lower quantile for body arch (Bar).
(F) *Z* score transformed firing rates (mean ± SEM, n = 52, 44, respectively, look-up and look-down units) as function of body arch (Bar, top panel) and overall motions (OM, bottom panel).
See also Figure S3.

CellPress
OPEN ACCESS

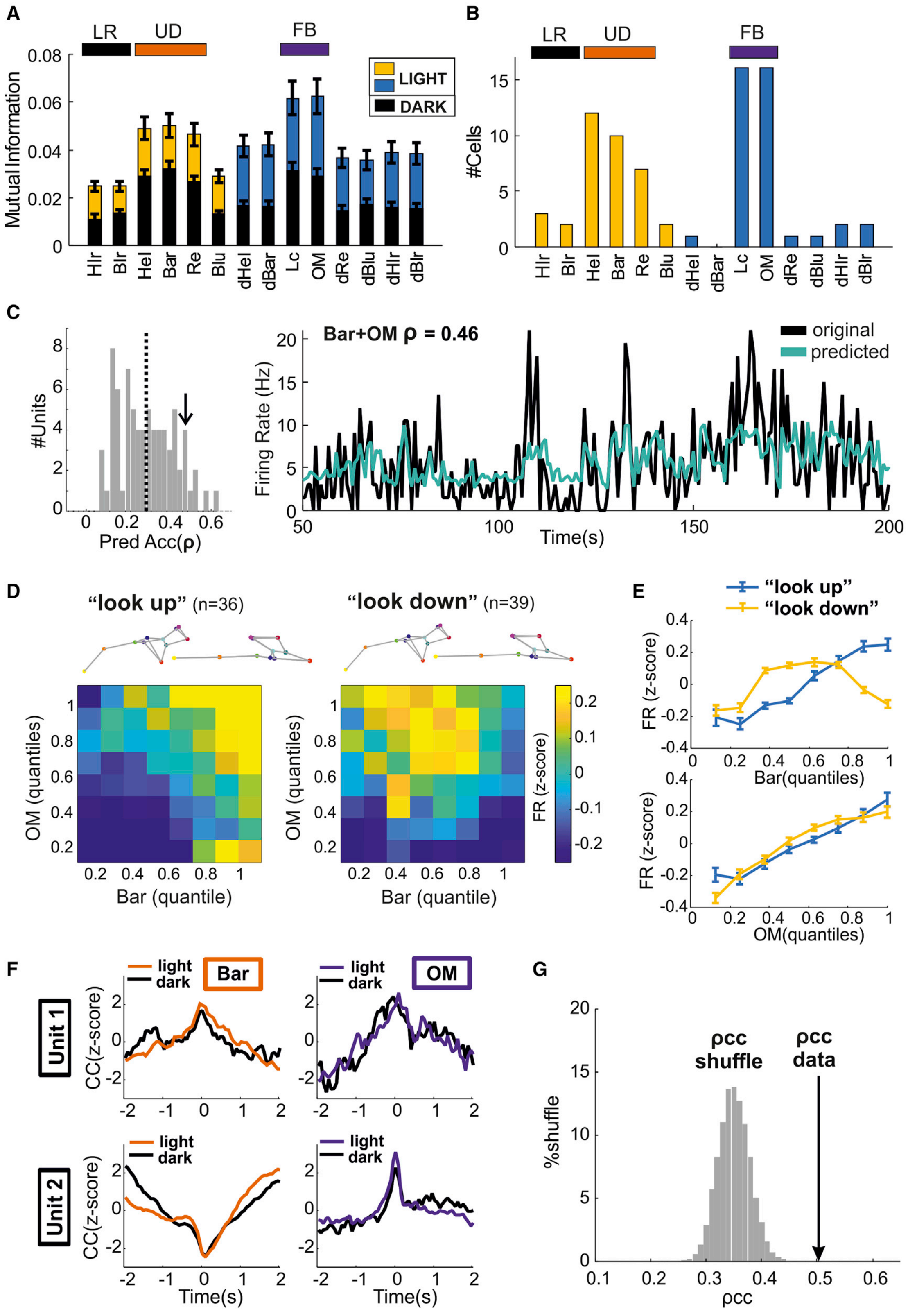

Figure 5. Modulation of single-unit activity by behavioral state is maintained and amplified by ambient illumination

(A) Mutual information (mean ± SEM, n = 75 units from 8 mice) for each motor state variable in dark and illuminated environments. Yellow and blue bars indicate, respectively, postures and movements.

(legend continued on next page)

head-fixed animals, the nature this preparation did not allow for measuring head movements and head and body postures. Two recent studies, performed in freely moving animals, quantified the effect of head movements, but not of head and body postures, in primary visual cortex.[16,17] Our results indicate that the effect of head movements (e.g., left-right head turns) on firing rates might be present in dlGN but is significantly less prominent than the effect of upward- and downward-facing postures (see e.g., Figure 3A).

Our second result was that the firing rate coupling to upward- and downward-facing postures was largely independent from, and interacted with, the coupling to full-body movements (and typically locomotion). This result addresses a long-standing debate about the nature of behavioral modulation in dLGN. Neuromodulation of primary visual thalamocortical loops has been traditionally associated with the control of sleeping and arousal states.[26] More recent studies have shown that modulation of neuronal activity in these regions is related to both motor activity and arousal (as measured via pupil dilation).[27–29,43,44] Separating the arousal component from motor activity component has been traditionally difficult using head-fixed preparations since locomotion on a treadmill is strongly coupled to pupil dilation.[27,29,44,45] Thus, although intermediate levels of arousal can occur without locomotion,[28,41,46,47] locomotion always coincide with high pupil dilation.[42,43,47] Our experiments in freely moving animals reveal two largely independent modulations, respectively, by full-body movements and upward- and downward-facing postures. Thus, although locomotion was associated with a generalized increased in firing rate concomitant with high arousal, this effect was gated by upward- and downward-facing postures, so that some units were most active when animals pointed their head high ("look-up" units), whereas others when animals kept their head low ("look-down" units). This result indicates that neuronal modulation in dLGN can be behavioral specific (e.g., some neurons will be active while exploring the ground, others while searching the sky) rather than simply reflecting a generalized state of arousal or motor activity. Additional experiments in other primary sensory thalamic regions will be required to understand whether these results also apply to other sensory modalities.

Our final result was that when experiments were repeated in an illuminated environment, the coupling of firing rate to postures and movements was maintained and amplified. The most parsimonious explanation is that the amplification would arise from the introduction of movement-related visual stimuli produced by self-motion. Alternatively, ambient light could drive neuronal activity of the visual thalamus into a more excitable state,[25,48–50] and this could amplify the coupling observed in the dark. Finally, if the coupling between firing rates and behavior is inherited from the cortex, ambient light could modify the interactions between excitatory and inhibitory populations in primary visual cortex[16,51] and, in turn, the corticofugal feedback onto the visual thalamus.

The sources of coupling to the behavioral state on the primary visual thalamus are currently unknown. Modulation by upward- and downward-facing postures could be provided by vestibular afferents from the brainstem, since optogenetic stimulation of the medial vestibular nucleus diffusely excites sensory thalamic nuclei and cortices.[52] Consistently, with this possibility, results from anesthetized cats showed that visual responses of >80% neurons in the dLGN were modulated by electrical stimulation of vestibular nuclei in the brainstem.[53] Primary visual cortex could also play an important role, by conveying both postural and motor information via the extensive direct and indirect (via TRN) corticofugal projections.[18] Strong inputs from thalamic regions other than TRN are unlikely, given the sparse intra-thalamic connectivity.[54] Visual thalamus has also been shown to receive afferents from the superior colliculus[20] and parabigeminal nucleus,[21] two important regions involved in visuomotor behaviors and action selection.[55] Additionally, direct or indirect input from the mesencephalic locomotor region (pedunculo pontine nucleus, laterodorsal tegmental nucleus) could be involved.[21,56] Finally, recent studies also provided evidence that arousal and locomotion modulate input from the retinal afferents[27,28] and that this modulation affects sensory processing in visual thalamus. Further studies will be needed to investigate the relative contribution of those sources.

Our results indicate that most neurons in visual thalamus are modulated according to multiple components of the ongoing behavior. This is consistent with recent studies of brain-wide modulation in mouse and flies and worms (for review, see Kaplan and Zimmer[57]), indicating that spontaneous neuronal activity is high dimensional and captures many distinct components of spontaneous behavior. However, a recent study compared the correlation between firing rate and running in marmoset and mouse primary visual cortices and found significantly smaller correlations in marmosets.[58] We currently do not understand the functional role(s) of brain-wide representation of behavior. One possibility is that excitation of specific subsets of neurons would provide a flexible encoding scheme to re-purpose visual processing according to ongoing behavior. Indeed, locomotion has been shown to modulate the gain of visual responses,[12,59] and this modulation can selectively amplify specific visual features (e.g.,

(B) Number of units best associated with each behavioral state variable according to our mutual information analysis (full-body movements, FB; up/down postures, UD; and left/right postures, LR, are highlighted by color rectangles at the top).
(C) Distribution of prediction accuracy for all units recorded (left panel, n = 75 from 8 mice; average accuracy shown as vertical dashed line). The arrow indicates a unit whose prediction based on body arch (Bar) and overall motion (OM) is shown on the right panel (black, original firing rate; green, predicted rate).
(D) *Z* score transformed firing rate (color coded) as function of body arch (Bar) and overall motion (OM). The units are divided into "look-up" and "look-down" units. The two poses at the top of each panel represent the extreme upper and lower quantile for body arch (Bar).
(E) *Z* score transformed firing rates (mean ± SEM, n = 36, 39, respectively, look-up and look-down units) as function of body arch (Bar, top panel) and overall motions (OM, bottom panel).
(F) Two example units in which we calculated cross-correlation (CC) of firing activity with body arch (Bar, left panels) and overall movements (OM, right panels). A strong similarity between CC calculated under dark (black lines) and light conditions (colored lines) is revealed for both units.
(G) Pearson's correlation between cross-correlation estimates in dark and light conditions (ρcc). The average Pearson's correlation (ρcc data) is significantly larger than values expected by randomly shuffling the pairing between units in dark and light (ρcc shuffle, n = 10,000 shuffles).
See also Figure S4.

transient ON responses[42]). Here, we show that in a freely moving animal, modulation is richer than just locomotion and different neurons are associated diverse visuomotor contingencies, indicating higher levels of flexibility. Consistent with our results, eye movements, largely suppressed in head-fixed preparations, are strongly driven by changes in upward- and downward-facing postures in freely moving animals.[60] The richer modulations observed in freely moving conditions could also be employed to support coordinate transformation and spatial navigation during spontaneous exploration[13,61] or to learn new visuomotor contingencies by gating visual inputs according to behavioral context.[62] Finally, behavioral modulation could be part of an encoding scheme that predicts incoming visual inputs based on self-motion.[14] These hypotheses are not mutually exclusive.

In summary, this study fills a gap in our understanding of how neuronal activity is coupled to behavior in the early visual system in natural unrestrained conditions. Our results indicate that neuronal activity in primary visual thalamus can be flexibly modulated according to movements and specific postures. The extent to which these modulations are applied to different stages of the visual pathway (and to other sensory pathways) is largely unknown. We believe that further investigation on this topic constitutes an important avenue for future studies.

STAR★METHODS

Detailed methods are provided in the online version of this paper and include the following:

- KEY RESOURCES TABLE
- RESOURCE AVAILABILITY
  - Lead contact
  - Materials availability
  - Data and code availability
- EXPERIMENTAL MODEL AND SUBJECT DETAILS
  - Animals
  - Ethical statement
- METHOD DETAILS
  - Recovery surgery
  - Experimental Set-Up
  - Behavioural protocol
- QUANTIFICATION AND STATISTICAL ANALYSIS
  - Reconstruction of 3D Poses
  - Quantification of behavioural state variables
  - Hierarchical clustering
  - Pre-processing of neuronal data
  - Cross-correlation analyses
  - Mutual information analyses
  - Predictive modelling analyses
  - Clustering of firing rate histograms

SUPPLEMENTAL INFORMATION

Supplemental information can be found online at https://doi.org/10.1016/j.cub.2022.07.049.

ACKNOWLEDGMENTS

We wish to express our gratitude to James Morizio and Harry Fu for support with TBSI recording system and to Annette Allen for comments and advice on the manuscript. This study was funded by a Sir Henry Dale fellowship from Wellcome Trust to R.S. (grant code: 220163/Z/20/Z); a David Sainsbury Fellowship from National Centre for Replacement, Refinement, and Reduction of Animals in Research (NC3Rs) to R.S. (grant code: NC/P001505/1); the European Union's Horizon 2020 Research and Innovation Programme under the Marie Sklodowska-Curie grant to P.O.-F (grant code: 897951); a Bekker Programme grant implemented by the Polish National Agency for Academic Exchange to P.O.-F. (grant code: PPN/BEK/2018/1/00192); a Wellcome Trust Investigator award (210684/Z/18/Z) to R.J.L; a research grant by Biotechnology and Biological Sciences Research Council to R.S.P. and R.S. (BBSRC; grant code: BB/V009680/1); and a grant from Weizmann – UK Making Connections Programme to R.S.P.

AUTHOR CONTRIBUTIONS

R.S., P.O.-F., and R.J.L. designed the study; R.S. and P.O.-F. performed experiments; R.S., P.O.-F., A.S.E., and A.G.Z. provided source codes and analyzed the data; and R.S., R.S.P., and R.J.L. wrote the manuscript.

DECLARATION OF INTERESTS

The authors declare no competing interests.

Received: March 2, 2022
Revised: May 24, 2022
Accepted: July 20, 2022
Published: August 15, 2022

REFERENCES

1. Land, M., Mennie, N., and Rusted, J. (1999). The roles of vision and eye movements in the control of activities of daily living. Perception *28*, 1311–1328. https://doi.org/10.1068/p2935.
2. De Franceschi, G., Vivattanasarn, T., Saleem, A.B., and Solomon, S.G. (2016). Vision guides selection of freeze or flight defense strategies in mice. Curr. Biol. *26*, 2150–2154. https://doi.org/10.1016/j.cub.2016.06.006.
3. Hoy, J.L., Yavorska, I., Wehr, M., and Niell, C.M. (2016). Vision drives accurate approach behavior during prey capture in laboratory mice. Curr. Biol. *26*, 3046–3052. https://doi.org/10.1016/j.cub.2016.09.009.
4. Goodale, M.A., and Milner, A.D. (1992). Separate visual pathways for perception and action. Trends Neurosci. *15*, 20–25. https://doi.org/10.1016/0166-2236(92)90344-8.
5. Cao, L., and Händel, B. (2019). Walking enhances peripheral visual processing in humans. PLoS Biol. *17*, e3000511. https://doi.org/10.1371/journal.pbio.3000511.
6. Miller, E.F., 2nd, Fregly, A.R., and Graybiel, A. (1968). Visual horizontal-perception in relation to otolith-function. Am. J. Psychol. *81*, 488–496.
7. Ganel, T., and Goodale, M.A. (2003). Visual control of action but not perception requires analytical processing of object shape. Nature *426*, 664–667. https://doi.org/10.1038/nature02156.
8. Jackson, S.R., and Husain, M. (2006). Visuomotor functions of the posterior parietal cortex. Neuropsychologia *44*, 2589–2593. https://doi.org/10.1016/j.neuropsychologia.2006.08.002.
9. Whitlock, J.R. (2014). Navigating actions through the rodent parietal cortex. Front. Hum. Neurosci. *8*, 293. https://doi.org/10.3389/fnhum.2014.00293.
10. Blot, A., Roth, M.M., Gasler, I., Javadzadeh, M., Imhof, F., and Hofer, S.B. (2021). Visual intracortical and transthalamic pathways carry distinct information to cortical areas. Neuron *109*, 1996–2008.e6. https://doi.org/10.1016/j.neuron.2021.04.017.
11. Wurtz, R.H., McAlonan, K., Cavanaugh, J., and Berman, R.A. (2011). Thalamic pathways for active vision. Trends Cogn. Sci. *15*, 177–184. https://doi.org/10.1016/j.tics.2011.02.004.

12. Niell, C.M., and Stryker, M.P. (2010). Modulation of visual responses by behavioral state in mouse visual cortex. Neuron *65*, 472–479. https://doi.org/10.1016/j.neuron.2010.01.033.

13. Saleem, A.B., Ayaz, A., Jeffery, K.J., Harris, K.D., and Carandini, M. (2013). Integration of visual motion and locomotion in mouse visual cortex. Nat. Neurosci. *16*, 1864–1869. https://doi.org/10.1038/nn.3567.

14. Keller, G.B., Bonhoeffer, T., and Hübener, M. (2012). Sensorimotor mismatch signals in primary visual cortex of the behaving mouse. Neuron *74*, 809–815. https://doi.org/10.1016/j.neuron.2012.03.040.

15. Bennett, C., Arroyo, S., and Hestrin, S. (2013). Subthreshold mechanisms underlying state-dependent modulation of visual responses. Neuron *80*, 350–357. https://doi.org/10.1016/j.neuron.2013.08.007.

16. Bouvier, G., Senzai, Y., and Scanziani, M. (2020). Head movements control the activity of primary visual cortex in a luminance-dependent manner. Neuron *108*, 500–511.e5. https://doi.org/10.1016/j.neuron.2020.07.004.

17. Guitchounts, G., Masís, J., Wolff, S.B.E., and Cox, D. (2020). Encoding of 3D head orienting movements in the primary visual cortex. Neuron *108*, 512–525.e4. https://doi.org/10.1016/j.neuron.2020.07.014.

18. Sherman, S.M., and Guillery, R.W. (2002). The role of the thalamus in the flow of information to the cortex. Philos. Trans. R. Soc. Lond. B Biol. Sci. *357*, 1695–1708. https://doi.org/10.1098/rstb.2002.1161.

19. Bickford, M.E., Slusarczyk, A., Dilger, E.K., Krahe, T.E., Kucuk, C., and Guido, W. (2010). Synaptic development of the mouse dorsal lateral geniculate nucleus. J. Comp. Neurol. *518*, 622–635. https://doi.org/10.1002/cne.22223.

20. Bickford, M.E., Zhou, N., Krahe, T.E., Govindaiah, G., and Guido, W. (2015). Retinal and tectal "driver-like" inputs converge in the shell of the mouse dorsal lateral geniculate nucleus. J. Neurosci. *35*, 10523–10534. https://doi.org/10.1523/JNEUROSCI.3375-14.2015.

21. Sokhadze, G., Whyland, K.L., Bickford, M.E., and Guido, W. (2022). The organization of cholinergic projections in the visual thalamus of the mouse. J. Comp. Neurol. *530*, 1081–1098. https://doi.org/10.1002/cne.25235.

22. Hubel, D.H. (1960). Single unit activity in lateral geniculate body and optic tract of unrestrained cats. J. Physiol. *150*, 91–104. https://doi.org/10.1113/jphysiol.1960.sp006375.

23. Jeczmien-Lazur, J.S., Orlowska-Feuer, P., Smyk, M.K., and Lewandowski, M.H. (2019). Modulation of spontaneous and light-induced activity in the rat dorsal lateral geniculate nucleus by general brain state alterations under urethane anesthesia. Neuroscience *413*, 279–293. https://doi.org/10.1016/j.neuroscience.2019.06.017.

24. Saalmann, Y.B., and Kastner, S. (2009). Gain control in the visual thalamus during perception and cognition. Curr. Opin. Neurobiol. *19*, 408–414. https://doi.org/10.1016/j.conb.2009.05.007.

25. Storchi, R., Bedford, R.A., Martial, F.P., Allen, A.E., Wynne, J., Montemurro, M.A., Petersen, R.S., and Lucas, R.J. (2017). Modulation of fast narrowband oscillations in the mouse retina and dLGN according to background light intensity. Neuron *93*, 299–307. https://doi.org/10.1016/j.neuron.2016.12.027.

26. Steriade, M., McCormick, D.A., and Sejnowski, T.J. (1993). Thalamocortical oscillations in the sleeping and aroused brain. Science *262*, 679–685. https://doi.org/10.1126/science.8235588.

27. Schröder, S., Steinmetz, N.A., Krumin, M., Pachitariu, M., Rizzi, M., Lagnado, L., Harris, K.D., and Carandini, M. (2020). Arousal modulates retinal output. Neuron *107*, 487–495.e9. https://doi.org/10.1016/j.neuron.2020.04.026.

28. Liang, L., Fratzl, A., Reggiani, J.D.S., El Mansour, O., Chen, C., and Andermann, M.L. (2020). Retinal inputs to the thalamus are selectively gated by arousal. Curr. Biol. *30*, 3923–3934.e9. https://doi.org/10.1016/j.cub.2020.07.065.

29. Erisken, S., Vaiceliunaite, A., Jurjut, O., Fiorini, M., Katzner, S., and Busse, L. (2014). Effects of locomotion extend throughout the mouse early visual system. Curr. Biol. *24*, 2899–2907. https://doi.org/10.1016/j.cub.2014.10.045.

30. Roth, M.M., Dahmen, J.C., Muir, D.R., Imhof, F., Martini, F.J., and Hofer, S.B. (2016). Thalamic nuclei convey diverse contextual information to layer 1 of visual cortex. Nat. Neurosci. *19*, 299–307. https://doi.org/10.1038/nn.4197.

31. Dunn, T.W., Marshall, J.D., Severson, K.S., Aldarondo, D.E., Hildebrand, D.G.C., Chettih, S.N., Wang, W.L., Gellis, A.J., Carlson, D.E., Aronov, D., et al. (2021). Geometric deep learning enables 3D kinematic profiling across species and environments. Nat. Methods *18*, 564–573. https://doi.org/10.1038/s41592-021-01106-6.

32. Zhang, L., Dunn, T., Marshall, J., Ölveczky, B.P., and Linderman, S. (2021). Animal pose estimation from video data with a hierarchical von Mises-Fisher-Gaussian model. Proceedings of the Machine Learning Research *130*, 2800–2808.

33. Nath, T., Mathis, A., Chen, A.C., Patel, A., Bethge, M., and Mathis, M.W. (2019). Using DeepLabCut for 3D markerless pose estimation across species and behaviors. Nat. Protoc. *14*, 2152–2176. https://doi.org/10.1038/s41596-019-0176-0.

34. Storchi, R., Milosavljevic, N., Allen, A.E., Zippo, A.G., Agnihotri, A., Cootes, T.F., and Lucas, R.J. (2020). A high-dimensional quantification of mouse defensive behaviors reveals enhanced diversity and stimulus specificity. Curr. Biol. *30*, 4619–4630.e5. https://doi.org/10.1016/j.cub.2020.09.007.

35. Chen, T., and Guestrin, C. (2016). KDD '16: Proceedings of the 22nd ACM SIGKDD International Conference on Knowledge Discovery and Data Mining (Association for Computing Machinery), pp. 785–794.

36. Benjamin, A.S., Fernandes, H.L., Tomlinson, T., Ramkumar, P., VerSteeg, C., Chowdhury, R.H., Miller, L.E., and Kording, K.P. (2018). Modern machine learning as a benchmark for fitting neural responses. Front. Comput. Neurosci. *12*, 56. https://doi.org/10.3389/fncom.2018.00056.

37. Newman, M.E. (2006). Finding community structure in networks using the eigenvectors of matrices. Phys. Rev. E Stat. Nonlin. Soft Matter Phys. *74*, 036104. https://doi.org/10.1103/PhysRevE.74.036104.

38. Stringer, C., Pachitariu, M., Steinmetz, N., Reddy, C.B., Carandini, M., and Harris, K.D. (2019). Spontaneous behaviors drive multidimensional, brain-wide activity. Science *364*, 255. https://doi.org/10.1126/science.aav7893.

39. Musall, S., Kaufman, M.T., Juavinett, A.L., Gluf, S., and Churchland, A.K. (2019). Single-trial neural dynamics are dominated by richly varied movements. Nat. Neurosci. *22*, 1677–1686. https://doi.org/10.1038/s41593-019-0502-4.

40. Mimica, B., Dunn, B.A., Tombaz, T., Bojja, V.P.T., and Whitlock, J.R. (2018). Efficient cortical coding of 3D posture in freely behaving rats. Science *362*, 584–589. https://doi.org/10.1126/science.aau2013.

41. Vinck, M., Batista-Brito, R., Knoblich, U., and Cardin, J.A. (2015). Arousal and locomotion make distinct contributions to cortical activity patterns and visual encoding. Neuron *86*, 740–754. https://doi.org/10.1016/j.neuron.2015.03.028.

42. Aydın, Ç., Couto, J., Giugliano, M., Farrow, K., and Bonin, V. (2018). Locomotion modulates specific functional cell types in the mouse visual thalamus. Nat. Commun. *9*, 4882. https://doi.org/10.1038/s41467-018-06780-3.

43. McCormick, D.A., Nestvogel, D.B., and He, B.J. (2020). Neuromodulation of brain state and behavior. Annu. Rev. Neurosci. *43*, 391–415. https://doi.org/10.1146/annurev-neuro-100219-105424.

44. Liu, D., and Dan, Y. (2019). A motor theory of sleep-wake control: arousal-action circuit. Annu. Rev. Neurosci. *42*, 27–46. https://doi.org/10.1146/annurev-neuro-080317-061813.

45. Reimer, J., Froudarakis, E., Cadwell, C.R., Yatsenko, D., Denfield, G.H., and Tolias, A.S. (2014). Pupil fluctuations track fast switching of cortical states during quiet wakefulness. Neuron *84*, 355–362. https://doi.org/10.1016/j.neuron.2014.09.033.

46. Lee, A.M., Hoy, J.L., Bonci, A., Wilbrecht, L., Stryker, M.P., and Niell, C.M. (2014). Identification of a brainstem circuit regulating visual cortical state in parallel with locomotion. Neuron *83*, 455–466. https://doi.org/10.1016/j.neuron.2014.06.031.

CellPress
OPEN ACCESS

47. McGinley, M.J., David, S.V., and McCormick, D.A. (2015). Cortical membrane potential signature of optimal states for sensory signal detection. Neuron *87*, 179–192. https://doi.org/10.1016/j.neuron.2015.05.038.

48. Storchi, R., Milosavljevic, N., Eleftheriou, C.G., Martial, F.P., Orlowska-Feuer, P., Bedford, R.A., Brown, T.M., Montemurro, M.A., Petersen, R.S., and Lucas, R.J. (2015). Melanopsin-driven increases in maintained activity enhance thalamic visual response reliability across a simulated dawn. Proc. Natl. Acad. Sci. USA *112*, E5734–E5743. https://doi.org/10.1073/pnas.1505274112.

49. Busse, L., Cardin, J.A., Chiappe, M.E., Halassa, M.M., McGinley, M.J., Yamashita, T., and Saleem, A.B. (2017). Sensation during active behaviors. J. Neurosci. *37*, 10826–10834. https://doi.org/10.1523/JNEUROSCI.1828-17.2017.

50. Lucas, R.J., Allen, A.E., Milosavljevic, N., Storchi, R., and Woelders, T. (2020). Can we see with melanopsin? Annu. Rev. Vis. Sci. *6*, 453–468. https://doi.org/10.1146/annurev-vision-030320-041239.

51. Dipoppa, M., Ranson, A., Krumin, M., Pachitariu, M., Carandini, M., and Harris, K.D. (2018). Vision and locomotion shape the interactions between neuron types in mouse visual cortex. Neuron *98*, 602–615.e8. https://doi.org/10.1016/j.neuron.2018.03.037.

52. Leong, A.T.L., Gu, Y., Chan, Y.S., Zheng, H., Dong, C.M., Chan, R.W., Wang, X., Liu, Y., Tan, L.H., and Wu, E.X. (2019). Optogenetic fMRI interrogation of brain-wide central vestibular pathways. Proc. Natl. Acad. Sci. USA *116*, 10122–10129. https://doi.org/10.1073/pnas.1812453116.

53. Papaioannou, J.N. (1972). Electrical stimulation of vestibular nuclei: effects on light-evoked activity of lateral geniculate nucleus neurones. Pflugers Arch. *334*, 129–140. https://doi.org/10.1007/BF00586786.

54. Swanson, L.W., Sporns, O., and Hahn, J.D. (2019). The network organization of rat intrathalamic macroconnections and a comparison with other forebrain divisions. Proc. Natl. Acad. Sci. USA *116*, 13661–13669. https://doi.org/10.1073/pnas.1905961116.

55. Shang, C., Chen, Z., Liu, A., Li, Y., Zhang, J., Qu, B., Yan, F., Zhang, Y., Liu, W., Liu, Z., et al. (2018). Divergent midbrain circuits orchestrate escape and freezing responses to looming stimuli in mice. Nat. Commun. *9*, 1232. https://doi.org/10.1038/s41467-018-03580-7.

56. Parent, M., and Descarries, L. (2008). Acetylcholine innervation of the adult rat thalamus: distribution and ultrastructural features in dorsolateral geniculate, parafascicular, and reticular thalamic nuclei. J. Comp. Neurol. *511*, 678–691. https://doi.org/10.1002/cne.21868.

57. Kaplan, H.S., and Zimmer, M. (2020). Brain-wide representations of ongoing behavior: a universal principle? Curr. Opin. Neurobiol. *64*, 60–69. https://doi.org/10.1016/j.conb.2020.02.008.

58. Liska, J.P., Rowley, D.P., Nguyen, T.T.K., Muthmann, J.-O., Butts, D.A., Yates, J.L., and Huk, A.C. (2022). Running modulates primate and rodent visual cortex via common mechanism but quantitatively distinct implementation. Preprint at bioRxiv. https://doi.org/10.1101/2022.06.13.495712.

59. Dadarlat, M.C., and Stryker, M.P. (2017). Locomotion enhances neural encoding of visual stimuli in mouse V1. J. Neurosci. *37*, 3764–3775. https://doi.org/10.1523/JNEUROSCI.2728-16.2017.

60. Meyer, A.F., O'Keefe, J., and Poort, J. (2020). Two distinct types of eye-head coupling in freely moving mice. Curr. Biol. *30*, 2116–2130.e6. https://doi.org/10.1016/j.cub.2020.04.042.

61. Salinas, E., and Thier, P. (2000). Gain modulation: a major computational principle of the central nervous system. Neuron *27*, 15–21. https://doi.org/10.1016/s0896-6273(00)00004-0.

62. Eren Sezener, A.G.-B., Kostadinov, D., Beau, M., Krishnagopal, S., Budden, D., Hutter, M., Veness, J., Botvinick, M., Clopath, C., Häusser, M., et al. (2021). A rapid and efficient learning rule for biological neural circuits. Preprint at bioRxiv. https://doi.org/10.1101/2021.03.10.434756.

63. Storchi, R., Rodgers, J., Gracey, M., Martial, F.P., Wynne, J., Ryan, S., Twining, C.J., Cootes, T.F., Killick, R., and Lucas, R.J. (2019). Measuring vision using innate behaviours in mice with intact and impaired retina function. Sci. Rep. *9*, 10396. https://doi.org/10.1038/s41598-019-46836-y.

64. Peirce, J.W. (2007). PsychoPy–Psychophysics software in python. J. Neurosci. Methods *162*, 8–13. https://doi.org/10.1016/j.jneumeth.2006.11.017.

65. Govardovskii, V.I., Fyhrquist, N., Reuter, T., Kuzmin, D.G., and Donner, K. (2000). In search of the visual pigment template. Vis. Neurosci. *17*, 509–528. https://doi.org/10.1017/s0952523800174036.

66. Jacobs, G.H., and Williams, G.A. (2007). Contributions of the mouse UV photopigment to the ERG and to vision. Doc. Ophthalmol. *115*, 137–144. https://doi.org/10.1007/s10633-007-9055-z.

67. XGBoost (2021). XGBoost documentation. https://xgboost.readthedocs.io/en/stable/.

68. Mathis, A., Mamidanna, P., Cury, K.M., Abe, T., Murthy, V.N., Mathis, M.W., and Bethge, M. (2018). DeepLabCut: markerless pose estimation of user-defined body parts with deep learning. Nat. Neurosci. *21*, 1281–1289. https://doi.org/10.1038/s41593-018-0209-y.

69. Cootes, T.F., Taylor, C.J., Cooper, D.H., and Graham, J. (1995). Active shape models-their training and application. Comput. Vision Image Underst. *61*, 38–59. https://doi.org/10.1006/cviu.1995.1004.

70. Tipping, M.E., and Bishop, C.M. (1999). Probabilistic principal component analysis. J. Royal Statistical Soc. B *61*, 611–622.

STAR★METHODS

KEY RESOURCES TABLE

REAGENT or RESOURCE	SOURCE	IDENTIFIER
Experimental Models: Organisms/Strains		
C576BL/6J	University of Manchester	RRID:MGI:5811150
Software and Algorithms		
MATLAB R2017a	The Mathworks	https://www.mathworks.com/products/matlab.html
Python	Python Software Foundation	https://www.python.org/
PsychoPy	Jonathan Peirce	http://www.psychopy.org/
Arduino	Arduino	http://www.arduino.cc
FlyCapture2	FLIR	https://www.flir.co.uk/products/flycapture-sdk/
Deposited Data		
Full 3D dataset of mouse poses; Full dataset of spiking activity; Code for the main analyses	Open Science Framework (OSF)	https://osf.io/q6cwp/

RESOURCE AVAILABILITY

Lead contact

Further information and requests for resources, reagents or raw data should be directed to and will be fulfilled by the Lead Contact, Riccardo Storchi (riccardo.storchi@manchester.ac.uk).

Materials availability

This study did not generate new unique reagents.

Data and code availability

Data and source codes are available at https://osf.io/q6cwp/. For additional information and support please contact riccardo.storchi@manchester.ac.uk.

EXPERIMENTAL MODEL AND SUBJECT DETAILS

Animals

Experiments were conducted on 12 adult, male C57BL/6J mice (Charles River). All mice were initially stored in cages of 5 individuals and housed individually after surgical implantation of the chronic electrode. Animals were provided with food and water *ad libitum* throughout their life and kept on a 12:12 light dark cycle.

Ethical statement

Experiments were conducted in accordance with the Animals, Scientific Procedures Act of 1986 (United Kingdom) and approved by the University of Manchester ethical review committee.

METHOD DETAILS

Recovery surgery

Throughout the procedure mice were anaesthetised with isoflurane (95/5% Oxygen/CO2 mix; flow rate: 0.5 – 1.0L/min). Concentrations of 4 – 5% and 0.5 – 1.5% were used respectively for induction and maintenance of anaesthesia. The level of anaesthesia was verified by the lack of withdrawal reflex. During the surgery animal's body temperature was automatically maintained at 37°C by a heating mat and animal's eyes were protected from drying out by eye drops. Once placed in the stereotaxic frame (Narishige, Japan), the animal fur was trimmed and 1% EMLA cream applied topically to the surrounding skin. An incision was made to expose a skull surface and set stereotactic *bregma* point, craniotomy and screws coordinates. Next, two slotted cheese machine screws (M1.6x2.0mm, Precision Tools, UK) were inserted respectively into the parietal, and interparietal plates to act as anchors for the dental cement and for electrical grounding of the electrode. After craniotomy the electrode was inserted into the dLGN (coordinates from *bregma*: 2.0-2.5mm medial-lateral, 2.3-2.5 mm rostro-caudal) at a depth of 2.8mm from the brain surface. To assess electrode

CellPress
OPEN ACCESS

placement we monitored light responses during surgical implantation of the electrode (Figure 5D). Light-curing cement (X-tra base, VO64434-A, VOCO) was applied to seal the implant. After surgery, the mouse was released from the ear bars and allowed to recover in a single-housed heated cage. Analgesia was provided with an intramuscular injection of 0.05mg/kg buprenorphine. After the procedure the animal was allowed to recover in a single-housed home-cage for a minimum of six days prior to experimentation. After all data were collected animals were perfused and histological post-mortem anatomy was performed to confirm electrode placement (Figure S5E).

Experimental Set-Up

A detailed description of the experimental set-up for behavioural recordings is provided in Storchi et al..[63] The animals were recorded in a square open field arena (dimensions: 30cm x 30 cm, Figure S1A). Behavioural recordings were acquired with 4 programmable cameras (Chamaleon 3 from Point Grey; frame rate = 15Hz) equipped with infrared cut-on filters (cut-on at 720 nm, Edmund Optics, #65-796) to isolate light in the infra-red range. Neuronal recordings were performed via 16 channel electrodes (Neuronexus; model: A4x4-3mm-50-125-177; package: CM16) with a TBSI W16 wireless acquisition system (Triangular BioSystems; sampling rate = 30 kHz). Frame acquisition, controlled via Psychopy (version 1.82.01, [64]), was synchronized with acquisition of neuronal data via an Arduino Uno board (www.arduino.cc). This board delivered a common electrical trigger to the cameras and the electrophysiological acquisition system.

The experiments were performed in light-tight cubicles that are part of the circadian facilities at the University of Manchester. Light-tightness is ensured by separating the experimental room from external light sources (such as windows) through 3 sealed doors that connect the cubicles to a corridor, the corridor to an antechamber and the antechamber to the rest of the facility. These measures were sufficient to bring light levels below the detection of any of our light meters (1.7nW; MACAM PM203 Optical Power Meter and SpectroCAL MKII Spectroradiometer) and of a dark-adapted human observer.

Behavioural protocol

Naïve animals were briefly anaesthetised (~2 minutes) with 3% isoflurane in order to connect the electrode head-stage. They were gently positioned at the centre of the arena and allowed plenty of time to recover from the anaesthesia. After the animals expressed sustained exploration of the arena (typically ~20 minutes after being placed in the arena) the experiment started. Animals were not specifically trained and freely explored the arena throughout the duration of the experiment (typically ~45 minutes). Arena illumination was through a rear projection screen mounted over the arena and provided excitation of $4.08*10^{10}$, $1.65*10^{13}$, $1.94*10^{13}$ and $2.96*10^{13}$ photon/cm^2/s respectively to S-cone opsin, Melanopsin, Rhodopsin and M-cone opsin. To calculate photon flux we weighted the spectral irradiance of our illumination by the pigment spectral efficiency estimated by using Govardovskii nomograms.[65] We then multiplied this by spectral lens transmission, measured in,[66] to account for the filtering effect of the mouse lens that attenuates light in the UV and near-UV band. During recordings in the dark, brief steps of light (0.66-1.33 seconds) were provided to test for visual responses. During recordings under steady illumination brief steps of dark (0.66-1.33 seconds) were also delivered. Video recordings were performed in epochs of 15-24 seconds, each separated by 30-40 seconds.

QUANTIFICATION AND STATISTICAL ANALYSIS

All analysis except the predictive modelling were performed in MATLAB (Natick, Massachusetts, USA) by using custom-made code. Predictive modelling of neuronal and behavioural data was performed in Python and based on xgboost library.[67]

Reconstruction of 3D Poses

An initial 3D reconstruction of the mouse body was obtained by triangulating body landmarks from the four cameras (see body landmarks in Figure 1A). The four-camera system was calibrated as previously described.[34] Tracking of body landmarks from individual cameras was performed with DeepLabCut.[68] The algorithm was trained with ~1000 manually annotated images and ran on a dedicated Ubuntu machine equipped with a Titan RTX GPU (Nvidia, Santa Clara, California, USA). The initial 3D reconstruction was contaminated with missing data and outliers, typically due to occluded body points. In order to correct the 3D reconstruction we modified a previously developed algorithm.[34] We first estimated a Statistical Shape Model (SSM, [69]) from a dataset of 350 manually re-annotated 3D poses. The poses were initially aligned by used Procrustes Superimposition (with scale parameter = 1). The SSM was then estimated by applying Probabilistic Principal Component Analysis (PPCA, [70]; MATLAB function: *ppca*). The SSM allowed us to express the 3D pose of the animal position as

$$\boldsymbol{X} = \left(\overline{\boldsymbol{X}} + \sum_{i=1}^{N_{eigenposes}} \boldsymbol{P}_i b_i\right)\boldsymbol{R} + \boldsymbol{T} \qquad \text{(Equation 1)}$$

Where: $\boldsymbol{X}$ is an N_p $x3$ matrix representing the 3D pose for given frame (N_p is the number of body landmarks); is the mean pose; $\boldsymbol{P}_i$ and b_i represent respectively the ith eigenpose obtained by training the SSM and its score (the shape parameter); $\boldsymbol{R}$ and $\boldsymbol{T}$ are respectively the $3\,x\,3$ rotation and N_p $x3$ translation matrices that map the animal's position in the experimental environment. Note that is $\boldsymbol{T}$ obtained as $\boldsymbol{T} = \boldsymbol{t} \otimes 1$ where $\boldsymbol{t} = [t_x, t_y, t_z]$ is the 3D translation vector and 1 is an x 1 all-ones vector. To obtain a robust 3D reconstruction we minimised, as function of $(\boldsymbol{b}, \boldsymbol{R}, \boldsymbol{T})$, the following cost function

$$C(\boldsymbol{b},\boldsymbol{R},\boldsymbol{T}) = \sigma^{-2}\left\|\boldsymbol{X} - \left(\overline{\boldsymbol{X}} + \sum_{i=1}^{N_{eigenposes}} b_i\boldsymbol{P}_i\right)\boldsymbol{R} - \boldsymbol{T}^2{}_F\right\| + \alpha\sum_{i=1}^{N_{eigenposes}}\frac{b_i^2}{\lambda_i} \quad \text{(Equation 2)}$$

Where: σ^2 represents the noise term obtained from the PPCA, λ_i the eigenvalue associated with the ith eigenpose and α, the regularization parameter, was set at 0.01. Outliers in a pose were flagged when the$C > inv(X^2(3N_p))$, with N_p indicting the number of body landmarks. When this happened, we removed the body landmark associated with largest value of C, reduced N_p by 1, and recalculated C. This operation was performed iteratively until $C < inv(X^2(3N_p))$. Then, in order to fill-up missing values and correct the remaining 3D coordinates, we recalculated $\boldsymbol{X}$ as in equation (1), by using ($\boldsymbol{b}$, $\boldsymbol{R}$, $\boldsymbol{T}$) values obtained from the minimization of equation (2) and $\overline{\boldsymbol{X}}$, $\boldsymbol{P}$ provided by the SSM.

Quantification of behavioural state variables

Quantification of behavioural state variables was based on 3D data after applying the reconstruction algorithms described above. Head elevation (Hel) was calculated as the angle between the nose and the neck landmarks. Head left and right turns (Hlr) as the angle between midline and nose. Body arch (Bar), body left and right turns (Blr), and body lounge (Blu) corresponded to the shape parameters associated respectively with first, second and third eigenposes. These definitions (e.g. body arch) were based on visual inspection of the videos in which we visualised the full body changes in shapes along each eigenpose (see Video S2). Rearing corresponded to the z-coordinate of the translation matrix $\boldsymbol{T}$. Locomotion (Lc) was calculated as Euclidean distance on the x-y axis of the body centre between two consecutive frames. The body centre in each frame was defined by the values of the translation vector $\boldsymbol{t}$ (see above). Overall motion (OM) was defined as the Euclidean across of all body points between two consecutive frames. All behavioural state variables where then transformed into z-scores for all further analyses.

Hierarchical clustering

Hierarchical agglomerative clustering across all behavioural state variables was applied to the Mutual Information matrix (Figure 1D). The hierarchical trees were created by using nearest distance method (MATLAB function: *linkage*, metric: *weighted*).

Pre-processing of neuronal data

Action potentials (typically >50 μV, see Figure 5A) were extracted from the continuous, high-pass filtered signals (low cut-off at 250 Hz) by using Offline Sorter (version 3). Noise artifacts were then removed. Then individual units were first sorted by combining template sorting method (Valley Seek) with Principal Component Analysis (PCA) in Offline Sorter software (Plexon, USA) and next manually adjusted. Inter-spike interval histograms were inspected to make sure that no spike events occurred during the refractory period (defined at 1ms duration). When there was more than one cluster on the channel (15 channels with 1 cluster and 58 channels with >1 cluster were spike sorted), reliable single unit isolation was confirmed by a MANOVA test (Offline Sorter). Signal-to noise ratio (SNR) was then calculated for each unit and only units with SNR>1.5 were kept for further analysis (Figures S5B and S5C). In order to avoid double counting the same unit from different channels we merged units that shared > 50% spikes timestamps from pairs of neighbouring channels (this only happened twice).

Cross-correlation analyses

Cross-correlation between spike counts and behavioural state variables was estimated after removal of the mean from each signal. In order to test for statistical significance we estimated a null-distribution by using a shuffling procedure. The association between spikes and behavioural state variables was broken by dividing spike counts into epochs and randomly permuting the order of such epochs. This operation was repeated 1000 times in order to generate the null-distribution. Original cross-correlation was deemed significant when at least one of its value (range [-2,+2] seconds; time-bin = 0.0667 seconds) was outside a [0.0005,0.9995] confidence interval.

Mutual information analyses

Prior to estimation, continuous behavioural state variables were transformed into discrete variables by applying quantile discretization. Probability distributions were then estimated from the frequency histograms of each signal. These distributions were used to obtain response and noise entropies, respectively calculated as

$$H(R) = -\sum_{g \in G} p(r)log_2p(r)$$

$$H(R|S) = -\sum_{g \in G,\, s \in S} p(r,s)log_2p(r|s)$$

Where: r indicates spike counts and s indicates the values of the behavioural state variables. Throughout this study we used a time-bin duration of 0.67 seconds for spike counts. Both entropy terms were corrected for the sampling bias by using quadratic extrapolation as in [63]. Shannon's Mutual Information (MI) was then calculated as $MI = H(R) - H(R|S)$. When comparing MI obtained from one vs two behavioural state variables (as e.g. in Figure 4A), we adopted the following strategy. First MI was estimated for two

CellPress
OPEN ACCESS

behavioural state variables as described above. Then, MI for single variables was estimated from the same two variables but by previously shuffling the order of one or of the other variable. Finally, the largest MI obtained by shuffling was taken as estimate of MI for an individual variable.

Predictive modelling analyses

Prediction of spike counts based on behavioural state variables was performed with eXtreme Gradient Boosting (XGBoost, [35],[67]; parameters: learning rate = 0.025; number of boosting iterations = 500; evaluation metric: log-likelihood loss; subsample = 1; maximum depth = 3; gamma = 1; tree method: gpu_hist). This method has been shown to outperform spike count predictions obtained with more standard approaches based on Generalized Linear Models.[36] Prediction of behavioural state variables from spike counts was also based on the same XGB models. All datasets were bisected into two equal parts that were used for training and cross-validation. Prediction performance on the cross-validation set were measured as Pearson's linear correlation between predicted and original firing rate. Throughout this study we used a time-bin duration of 0.67 seconds for spike counts. For prediction of spike counts, in order to test for the possibility that positive correlations were obtained purely by chance, we repeated this analysis by shifting the behavioral state variables. Shifting was obtained by splitting the behavioural state time series into two equally long epochs and inverting the order of those epochs. In this way the association of these time series with spike counts was abolished while the temporal structure of those series was preserved.

Clustering of firing rate histograms

We use Newman's algorithm for community detection, that maximizes network modularity.[37] The higher the modularity the better the clustering. For our data each unit of the network is a bivariate firing rate histogram calculated as function of body arch and overall motion. The adjacency matrix for the network was based on Spearman's correlation between firing rate histograms. Each pair of units was considered connected when their pairwise correlation was above the median the correlation distribution, The algorithm works by iteratively bisecting the network.[37] Therefore it first considers the whole network and bisects it according to the first eigenvector of the modularity matrix (eq 3 in Newman 2006[37]). Then it considers each partition and perform further bisections. The algorithm terminates when it cannot find any further bisection that would increase network modularity.

Current Biology, Volume *32*

Supplemental Information

Look-up and look-down neurons in the mouse visual thalamus during freely moving exploration

Patrycja Orlowska-Feuer, Aghileh S. Ebrahimi, Antonio G. Zippo, Rasmus S. Petersen, Robert J. Lucas, and Riccardo Storchi

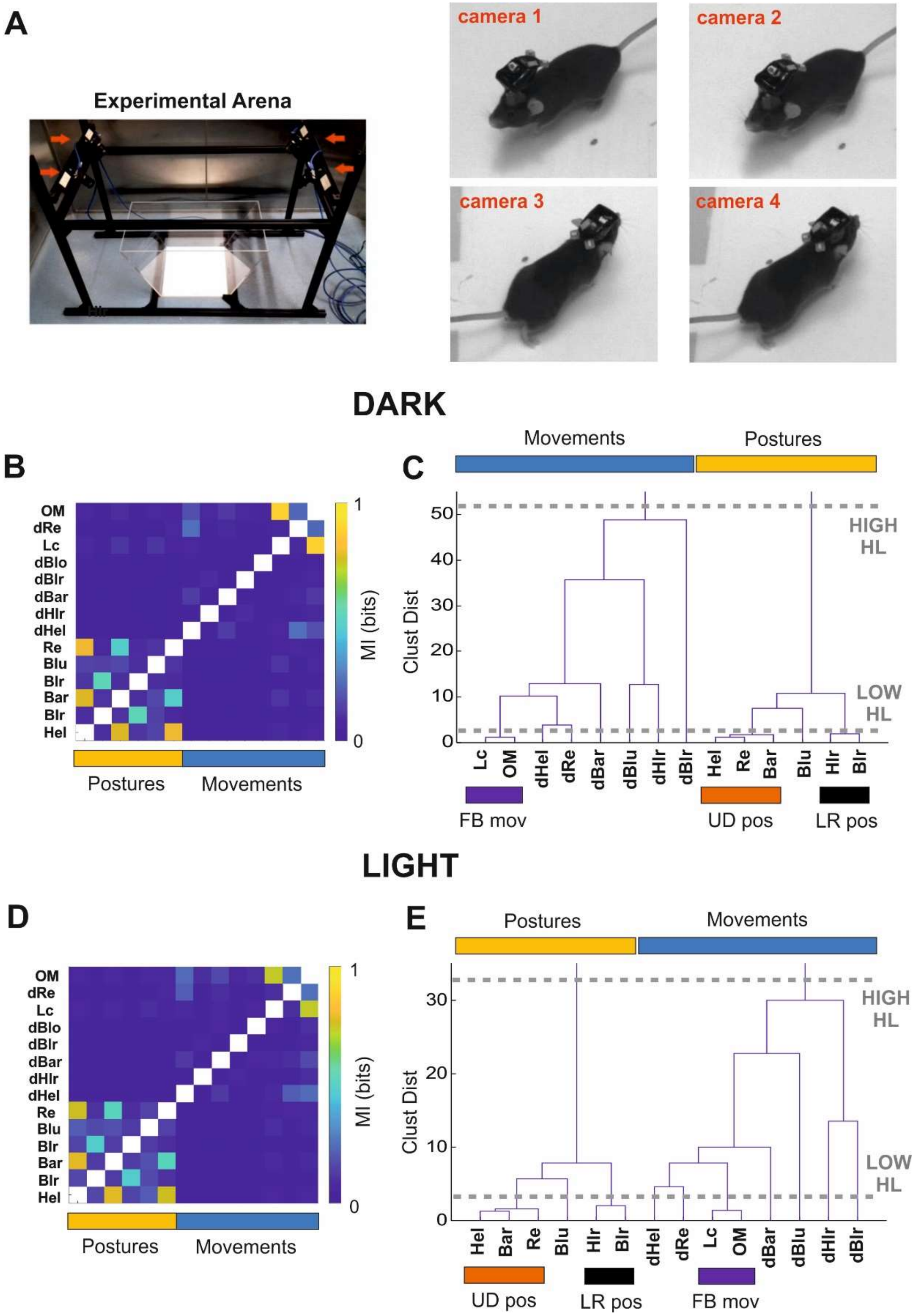

Figure S1. Behavioural set-up and supplemental analyses of behavioural state variables. Related to Figure 1: A) A pictures of the experimental apparatus (left panel). The arena is placed in the centre and imaged by four overhead cameras (indicated by red arrows). A representative frame simultaneously acquired by the 4 cameras is shown (right panel). **B-C)** Same as Figure **1C-D** but for animals recorded in the dark. **D-E)** Same as Figure 1C-D but for animals recorded under photopic light.

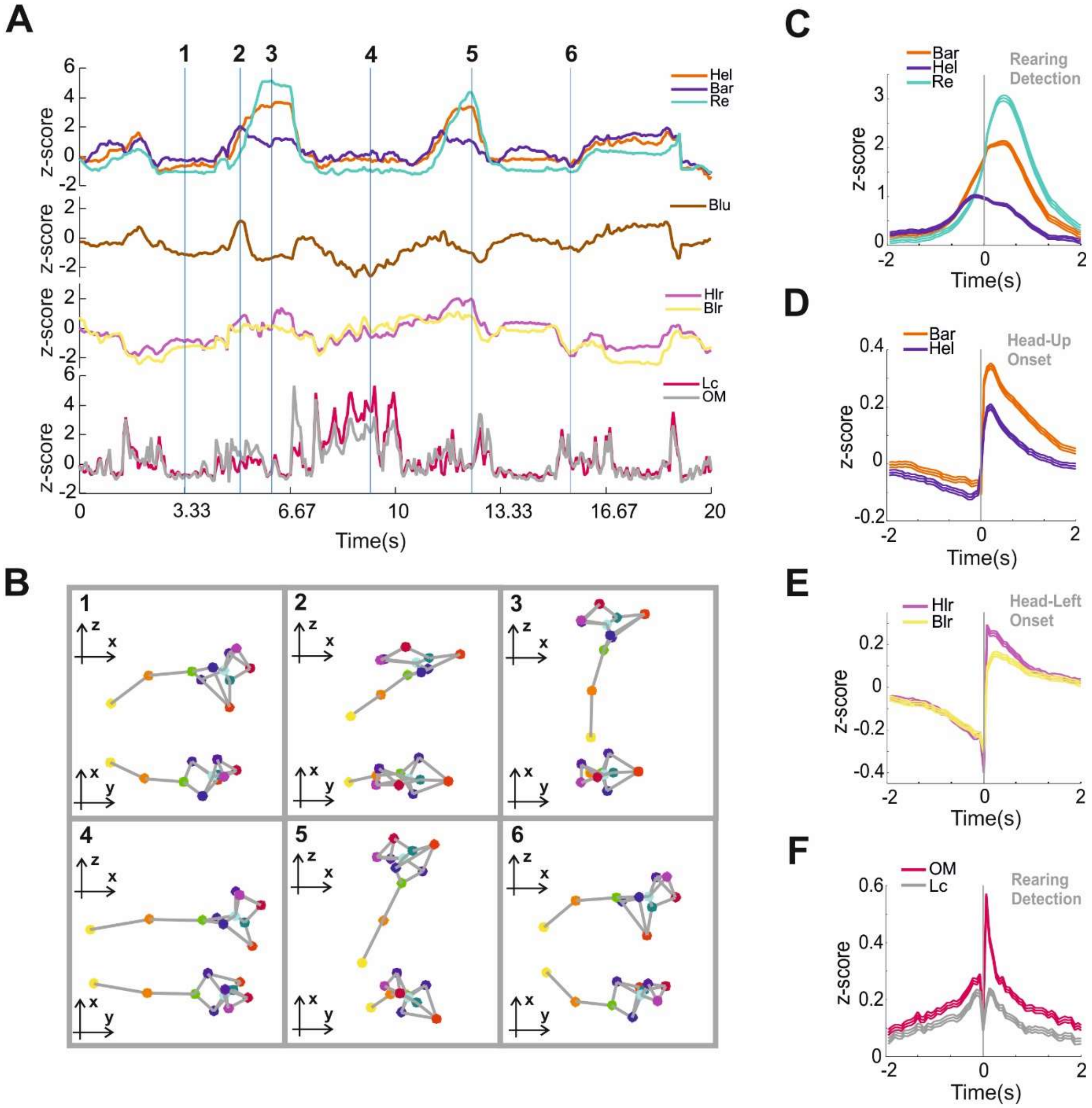

Figure S2. Supplemental analyses of behavioural state variables. Related to Figure 1: A) Representative time series for a selection of behavioural state variables (head elevation, Hel, body arch, Bar, rearing, Re, body lunge, Blu, head left/right, Hlr, body left/right, Blr, locomotion, Lc, overall motion, OM – the variables not shown are simply obtained as time derivative of the postures). **B)** Poses from six frames (indicated in panel A by vertical blue lines) are shown from side and top view. **C)** Average bodt arch (Bar), rearing (Re) and head elevation (Hel) at the onset of a rearing event (detected at time 0). Note that body arch and head elevation precede rearing. **D)** Average body arch (Bar) and head elevation (Hel) at the onset of an upward head movement (detected at time = 0). **E)** Same as panel D but for head left/right (Hlr) and body left/right (Blr) measured at onset of a leftward head movement. **F)** Average overall motion (OM) and locomotion (Lc) at the onset of a rearing event.

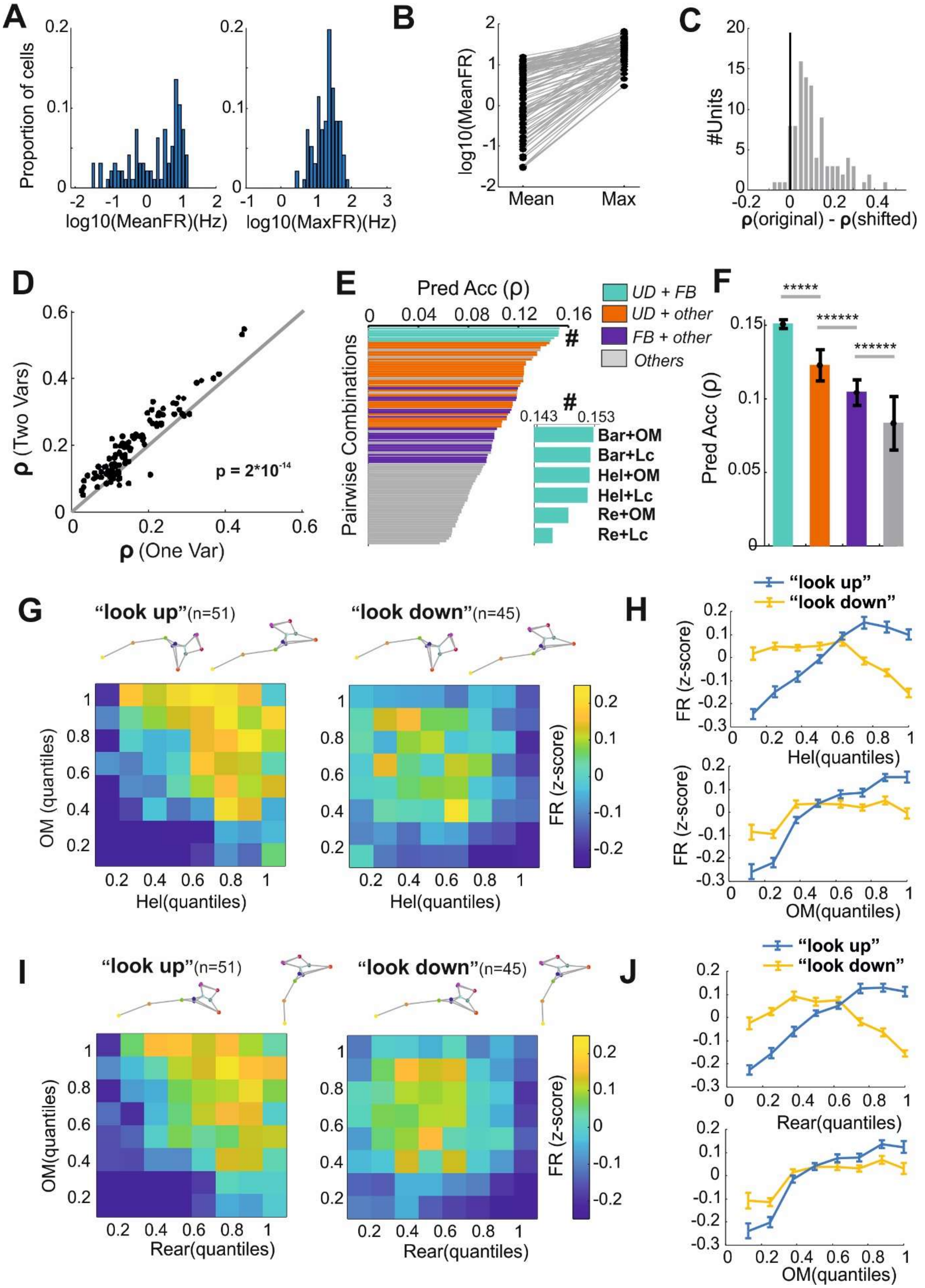
A
Proportion of cells
log10(MeanFR)(Hz)
log10(MaxFR)(Hz)
B
log10(MeanFR)
Mean
Max
C
#Units
ρ(original) - ρ(shifted)
D
ρ (Two Vars)
ρ (One Var)
p = 2*10^-14
E
Pred Acc (ρ)
Pairwise Combinations
UD + FB
UD + other
FB + other
Others
#
0.143
0.153
Bar+OM
Bar+Lc
Hel+OM
Hel+Lc
Re+OM
Re+Lc
F
Pred Acc (ρ)
*****
******
******
G
"look up"(n=51)
"look down"(n=45)
OM (quantiles)
Hel(quantiles)
FR (z-score)
H
"look up"
"look down"
FR (z-score)
Hel(quantiles)
OM(quantiles)
I
"look up"(n=51)
"look down"(n=45)
OM(quantiles)
Rear(quantiles)
FR (z-score)
J
"look up"
"look down"
Rear(quantiles)
OM(quantiles)

Figure S3. Supplemental analyses of coupling between behavioural state variables and neuronal activity in the dark. Related to Figure 2, Figure 3 and Figure 4: A) Distribution of mean (left panel) and peak firing rates (right panel) for our dataset (n = 96 units from 11 mice). **B)** Relation between mean and peak firing rates for the same dataset shown in panel A. **C)** Paired difference in prediction accuracy (measured as Person's ρ) between original and shifted predictors (n = 96 units for 11 mice). **D)** Comparison between prediction accuracy obtained with one variable (x-axis) and two variables (y-axis). **E)** Each horizontal bars indicates the average prediction accuracy for pairwise combinations of behavioural state variables (n = 96 units from 11 mice). Bars are colour-coded according to the type of variables (e.g. green for pairwise combinations of up/down postures and full body movements, see legend). The inset (indicated by #) magnifies the top six pairwise combinations. **F**) Prediction accuracy (Mean±SEM, n = 96 units from 11 mice) for each class of predictors. **G**) Z-score transformed firing rate (color coded) as function of head elevation (Hel) and overall motion (OM). The units are divided into "look-up" and "look-down" units. The two poses at the top of each panel represent the extreme upper and lower quantile for head elevation. **H**) Z-score transformed firing rates (mean±SEM, n = 52, 45, respectively look-up and look-down units) as function of head elevation (Hel, top panel) and overall motions (OM, bottom panel). **I-J)** Same as panels G-H but for rearing (Re) instead of head elevation.

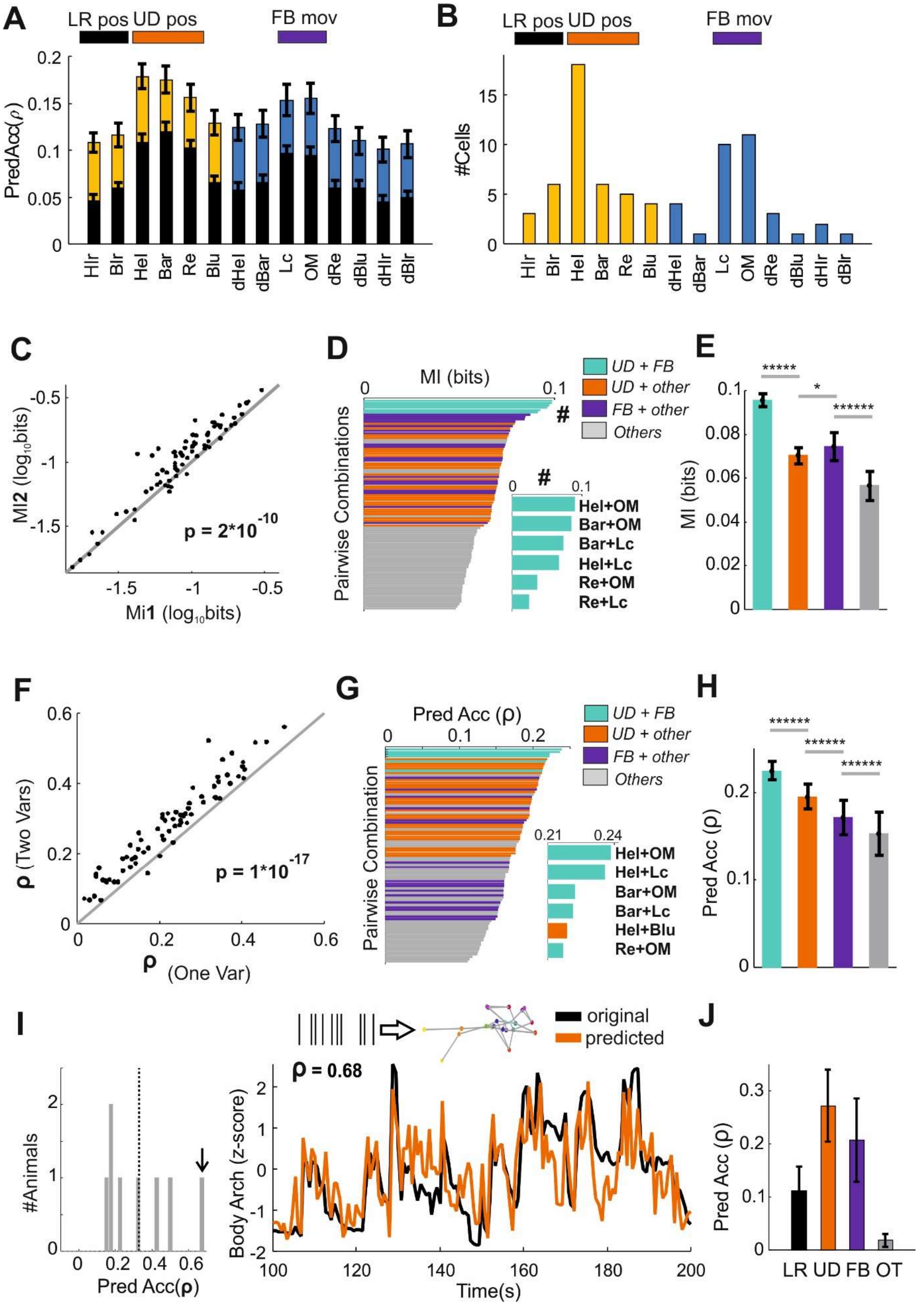

A
LR pos
UD pos
FB mov
PredAcc(ρ)
Hlr
Blr
Hel
Bar
Re
Blu
dHel
dBar
Lc
OM
dRe
dBlu
dHlr
dBlr
B
#Cells
C
MI2 (log10bits)
Mi1 (log10bits)
p = 2*10-10
D
MI (bits)
Pairwise Combinations
UD + FB
UD + other
FB + other
Others
Hel+OM
Bar+OM
Bar+Lc
Hel+Lc
Re+OM
Re+Lc
E
MI (bits)
F
ρ (Two Vars)
ρ (One Var)
p = 1*10-17
G
Pred Acc (ρ)
Pairwise Combination
Hel+OM
Hel+Lc
Bar+OM
Bar+Lc
Hel+Blu
Re+OM
H
Pred Acc (ρ)
I
#Animals
Pred Acc(ρ)
original
predicted
ρ = 0.68
Body Arch (z-score)
Time(s)
J
Pred Acc (ρ)
LR
UD
FB
OT

Figure S4. Supplemental analyses of coupling between behavioural state variables and neuronal activity under photopic light. Related to Figure 5: A) Prediction accuracy (mean±SEM, n = 75 units from 8 mice) for each behavioural state variable in dark and illuminated environments (indicated respectively by black and coloured bars). **B)** Number of units best associated with each behavioural state variable according to our prediction analysis (full body movements, FB, upward/downward facing postures, UD, and left/right postures, LR, are highlighted by colour rectangles at the top). **C)** Comparison between Mutual Information (MI) conveyed by one predictor (x-axis; MI calculated as shuffle control) and two predictors (y-axis). **D)** Each horizontal bar indicates the average MI between firing rates (n = 75 units from 8 mice) and a pairwise combination of behavioural state variables. Bars are colour-coded according to the type of variables (e.g. green for up/down postures and full body movements, UD+FB, see legend). The inset (indicated by #) magnifies the top six pairwise combinations. **E**) Mutual Information (Mean±SEM, n = 75 units from 8 mice) for each class of predictors. **F-H)** Same as panels C-E but here we calculated prediction accuracy instead of Mutual Information. **I)** Distribution of prediction accuracy for all mice (n = 11; average accuracy shown as vertical dashed line). The arrow indicates an animal whose prediction based on body arch (Bar) is shown on the right panel (black = original firing rate; orange = predicted rate). **J**) Average prediction accuracy (Mean±SEM) for all animals (n=8) as function of left/right postures (LR), up/down postures (UD), full body movements (FB) and other variables (OT, which indicates the remaining 7 variables).

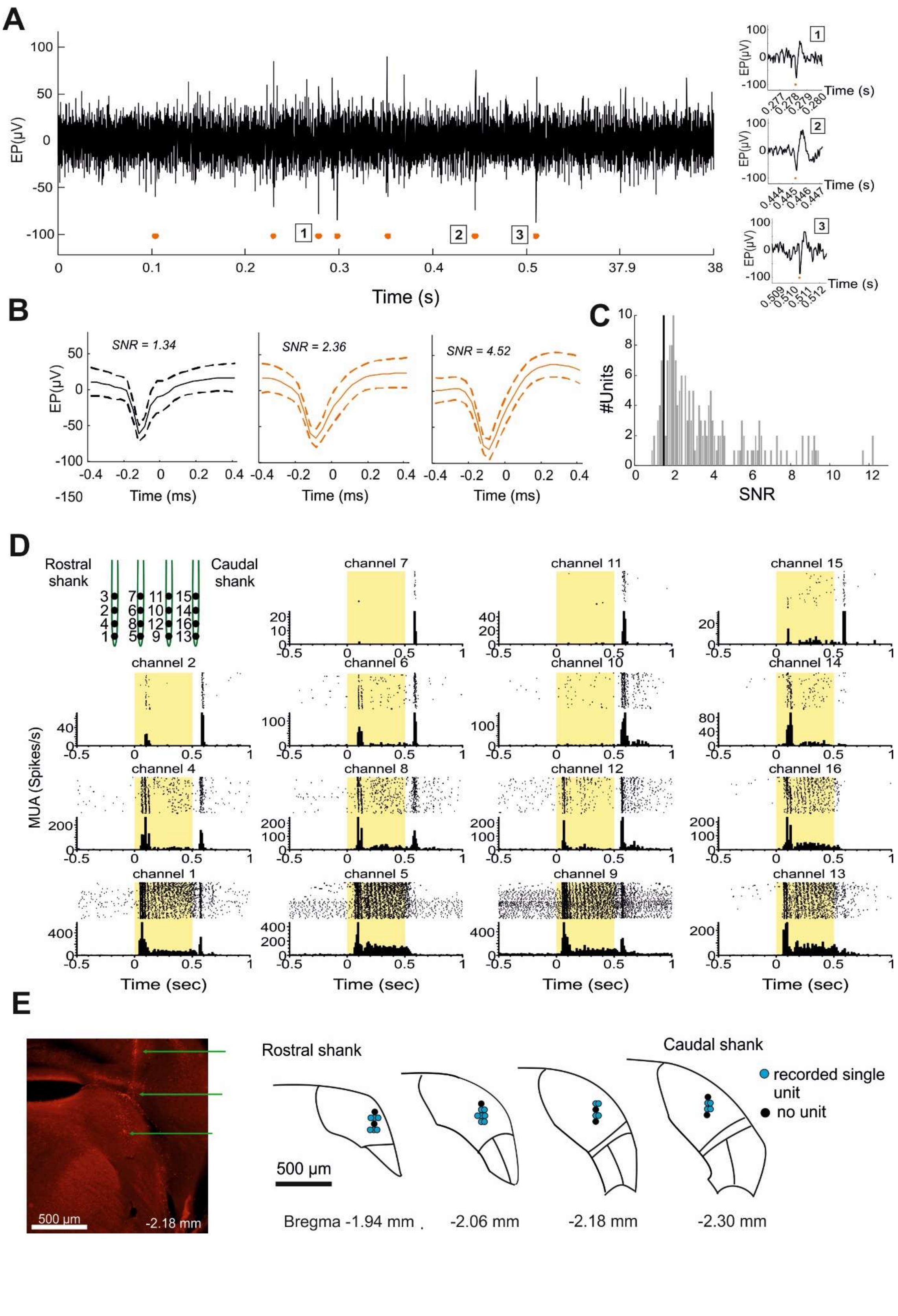
A
EP(μV)
Time (s)
1
2
3
B
SNR = 1.34
SNR = 2.36
SNR = 4.52
Time (ms)
C
#Units
SNR
D
Rostral shank
Caudal shank
channel 1
channel 2
channel 3
channel 4
channel 5
channel 6
channel 7
channel 8
channel 9
channel 10
channel 11
channel 12
channel 13
channel 14
channel 15
channel 16
MUA (Spikes/s)
Time (sec)
E
500 μm
-2.18 mm
Rostral shank
Caudal shank
recorded single unit
no unit
Bregma -1.94 mm
-2.06 mm
-2.18 mm
-2.30 mm

Supplementary Figure 5. Extracellular recordings and anatomical localization. Related to Figure 1 and STAR Methods: A) Representative extracellular recording. Spikes detected are indicated by orange dots and three of them magnified for visual inspection (see insets 1-3). **B)** Representative sample of recorded units. Signal-to-Noise ratio (SNR) is reported at the top of each panel. Only units with SNR>1.5 were used for further analyses (here we excluded the first unit on the left since SNR=1.34). **C)** Distribution of SNR across our dataset (black vertical line indicates the threshold for inclusion). **D)** Visual responses from extracellular recordings at the end of surgical implantation of the recording electrodes. Yellow shading indicates the epoch in which full-field light stimulation was on (light onset at time 0, stimulus duration = 0.5s). Top-left panel shows the multichannel electrode layout. **E)** Histological verification of electrode placement in which fluorescent trace indicate the position of the rostral shank (left panel). Right panel shows the estimated position of isolated single units (blue dots) along rostro-caudal axis.
